# The potential contribution of citizen science data in the study of coastal microplastic and mesoplastic distributions

**DOI:** 10.1007/s10661-025-14354-2

**Published:** 2025-07-21

**Authors:** David M. Jones, Jonathan Potts, Michelle S. Hale

**Affiliations:** School of the Environment and Life Sciences, University of Portsmouth, King Henry Building, Portsmouth, PO1 2DY UK

**Keywords:** Microplastics, Mesoplastics, Coastal, Citizen science

## Abstract

Plastic pollution in marine environments is an escalating global issue, particularly in coastal regions where geomorphic processes and relatively high waste input can lead to significant accumulation. In this study, we analysed citizen science (CS) data from the Big Microplastic Survey (BMS), collected by volunteers across 39 countries between March 2018 and April 2024. The BMS project provided information about microplastic (MP) and mesoplastic (MEP) distributions from coastlines around the world. Using descriptive statistics, we examined volunteer participation and engagement, as well as the geographical distribution of MPs and MEPs based on characteristics and colour. Our analysis highlighted some of the challenges associated with CS projects and the importance of organisational affiliations in influencing data submission. There was substantial variability in the geographical distributions of plastics, suggesting regional pollution dynamics that were influenced by both anthropogenic and natural coastal processes. In this study, our analysis of global, regional, and localised data underscored the value of CS in augmenting traditional scientific methods, providing critical insights into coastal plastic pollution, and potentially engaging communities in environmental stewardship. We also discovered that engaging with non-governmental organisations (NGOs) was particularly effective in facilitating data collection. We advocate for a strategic focus on organisational participation in CS projects, to improve data collection and assess spatial and temporal trends more fully.

**Supplementary Information:**

The online version contains supplementary material available at 10.1007/s10661-025-14354-2.

## Introduction

Whilst the global production of plastic increases on an annual basis (Plastics Europe, 2022), the world continues to struggle to address the impact of plastic pollution in the marine environment. The problem is particularly noticeable in coastal areas where plastic pollution tends to accumulate through geomorphic processes and relatively high waste input, negatively impacting marine ecosystems, coastal communities, and economies (GESAMP, 2016; Brouwer et al., 2017). Growing awareness and concern have culminated in the adoption of an internationally recognised and legally binding resolution to end plastic pollution, including in the marine environment, by the United Nations Environment Assembly (UNEA) in 2022 (United Nations Environment Assembly, 2022). Amongst the provisions of this UNEA resolution is a requirement for member nations to provide scientific assessment related to plastic pollution and promote national action plans. In the European Union, the establishment of assessment and monitoring guidelines known as the Marine Strategy Framework Directive (MSFD) provided a framework for member states to implement programmes to collect scientific data (Hanke et al., 2013); similar guidelines are in operation in other parts of the world (Nyadjro et al., 2023). These management processes provide a mechanism for long-term monitoring, an essential element of understanding system dynamics and subsequently being able to influence policy aimed at reducing waste and pollution (Ducklow et al., 2009). However, in many countries, particularly in geographically remote areas and low and middle-income nations where plastic pollution is often more problematic (Browning et al., 2021), such programmes are limited. Gathering long-term data in these regions can be challenging (Giron-Nava et al., 2017; Jones et al., 2022), resulting in gaps in our understanding, and, consequently, delays in policy implementation. Some of those gaps can be addressed, in part, by citizen science (CS) research projects that have been developed because of the increasing awareness of numerous environmental issues (Bayas et al., 2017; Chandler et al., 2017; Tulloch et al., 2013). These CS projects are becoming increasingly recognised as a useful and cost-effective data resource to augment data collected by more traditional scientific methods (Nelms et al., 2017). Although some scepticism exists within the scientific community regarding the quality of CS data (Aceves-Bueno et al., 2017; Riesch & Potter, 2014; Wiggins et al., 2014) and there are concerns regarding inherent spatial and temporal bias that results from the opportunistic nature of CS projects (Callaghan et al., 2019; Millar et al., 2019), these citizen-based monitoring (CBM) studies can provide opportunities to examine spatial and temporal trends on a wider scale and in geographic locations that might otherwise not be sampled (Kelly et al., 2020; Le Rest et al., 2015).

We previously published on the development of one such CS-based approach known as the Big Microplastic Survey (BMS), which aimed to collect data on microplastics (MPs) and mesoplastics (MEPs) in the 1–25 mm size range in coastal areas around the world (Jones et al., 2024). Subsequently, we compared this approach with other methods including the MSFD protocol mentioned previously and found that there were statistically significant differences in the results obtained between differing methods in some circumstances and concluded that standardisation would remain a challenge (Jones, et al., 2025). Following its roll-out in an open-access format in 2018, the BMS project has allowed volunteers from around the world to submit data to an online portal. Since the project launch, participation has been widespread, with a total of 1089 surveys being submitted from 39 countries or independent territories. In this study, we explore the wealth of submitted data from the BMS project in order to assess and discuss the application of CS in the study of coastal MP and MEP by (1) analysing volunteer registration and participation data; (2) exploring the total count and characteristics of samples based on geographical distribution; (3) examining spatial and temporal trends in specific areas where sufficient data have been collected; and (4) discussing the application and use of CS in supporting more traditional long-term monitoring and data collection programmes.

## Methodology

### Sample dataset

The dataset for this study comprised CS researchers’ (participants’) project registration information as well as MP and MEP data submitted during surveys undertaken using the BMS sampling methodology (Jones et al., 2024), between March 2018 and April 2024. A prerequisite of participation was a requirement to register on the project website. Once registered, volunteers were provided with a participant registration number that they used when completing a survey and submitting data. On completion of their surveys, volunteers uploaded their data onto the project website. This information was automatically transferred to an ESRI Global Information System (GIS) (https://www.esriuk.com) database which, in turn, populated an online, map-based results page that provides open-source information of each survey. Each survey was given a unique identification number by the software and the date of data submission was recorded automatically. During data submission, participants were able to include information about any organisational affiliations if applicable, a name/location for the survey site, as well as a description of the site and the number of volunteers involved. Participants also entered latitude and longitude information for each survey undertaken and the BMS project online supporting documentation provided guidance on how to do this. For each survey, participants entered numeric values for the number of plastic pieces found for a range of MP and MEP types and categories, subdivided into specific colours. The total volume of sediment collected in each sample using the BMS method was 0.005 m^3^. Participants were also required to submit an image of the plastic sample they had analysed, for verification purposes. The complete dataset of participant details and sample submissions as of 6 April 2024 was provided by the BMS project from their ESRI database.

### Data organisation

Initial data organisation involved reorganising the sample plastic characteristics into a readable format by importing the data into the programming language R version 4.0.3 (R Core Team, 2020) and the package Tidyverse (Wickham et al., 2019). Data organisation included summarising source (secondary/primary), type (nurdles/bio-beads^1^/expanded polystyrene (EPS)), shape (pre-production pellets only), size (MP or MEP), and colour as determined by the BMS methodology, along with total plastic counts for each category. In addition, participant organisational affiliations were divided into the following groups: non-government organisations (NGOs); scouting groups; schools/colleges; universities; companies (business entities); government agencies; and unaffiliated individuals.

A visual inspection of the survey locations using ESRI Global Information System (GIS) mapping software (https://www.esriuk.com) highlighted several obviously inaccurate survey locations. These locations appeared to have been uploaded incorrectly for two reasons: incorrect submissions by participants (for example confusing latitude and longitude) and an issue in the data upload site using ESRI Survey 123 (https://survey123.arcgis.com) that resulted in the data defaulting to a pre-determined location if coordinates were not submitted. Uhrin et al. (2022) found similar discrepancies in their study and adjusted sample site locations based on best evaluations. Using the site locations and descriptions that were submitted by the participants along with plastic characteristics during data collection, we were similarly able to adjust the coordinates of samples using best evaluation from the information available.

### Data analysis

The data were separated into categories to allow for the analysis of registration and participation information, as well as the geographical distribution and characteristics of MP and MEPs found in samples. The first analysis was an examination of the volunteer registration and participation details. Participants who submitted surveys were cross-referenced against the initial registration database to understand how an initial declaration of interest in the project translated into participation. Additionally, based on information supplied in the registration database, participants were affiliated to seven organisational categories that were identified: non-government organisations (NGOs), scouting groups, business entities (companies), schools/colleges, universities, government agencies, and individuals. This in turn enabled submitted data samples to be allocated to specific organisations. Anyone that did not submit an affiliation during the registration process was assumed to be an individual. Data regarding registration and participation were presented in a tabulated format, and analysis was undertaken using descriptive statistics.

The second data analysis was a broad global overview of the complete MP and MEP dataset based on the locations of samples at a country level and the characteristics of the plastics collected by the volunteers. Country identification based on those listed in ISO 3166 Alpha-2 codes (https://www.iso.org/iso-3166-country-codes.html) which included independent British Overseas Territories and Antarctica was used to be able to utilise the data within ESRI mapping software. The data were then tabulated to show average counts based on the number of samples in each country and explored using descriptive statistics and ESRI mapping software to provide visual representations of MP and MEP distribution based on national boundaries, sample type, and colour characteristics in this and subsequent analyses.

For the third analysis, data were divided into smaller regional groupings to enable a more detailed examination to be undertaken within specific countries. In their analysis of plastic waste in the USA, Milbrandt et al. (2022) divided their data into state, county, and local levels. This is relatively simple in countries where there is a well organised regional structure; however, it can become more complex, for example, when there are multiple countries within a sovereign state, as in the case of the UK; variations in types and nomenclature of regional structures; or undefined administrative boundaries. Where possible, we divided the country samples into sub-level regions based on the ‘next level down’ administrative area such as states in the USA, statistical regions in Portugal, provinces in Argentina and Mozambique, and governates in Bahrain. It was decided to explore the UK at a county level rather than divide it into the individual nations within Great Britain and Northern Ireland. Additionally, as the data from Greece, Honduras, Malaysia, and Palau were from offshore islands with similar biogeographical features, it was decided to sub-divide the data in these situations at an individual island level to provide insights into possible localised trends (Lavers et al., 2019; Vilhena & Antonelli, 2015). The offshore island of Koh Tao in Thailand was also identified as an independent sub-level region. Analysis at this regional level was undertaken on those countries that had five or more sub-level administrative areas. Programming language R version 4.0.3 (R Core Team, 2020) and the packages Tidyverse (Wickham et al., 2019) and ggplot2 (Wickham, 2016) were used in this and subsequent analyses to produce graphical representations showing MP and MEP characteristics to support the descriptive analysis.

In the final data analysis, we looked at the number of samples within each sub-region and the period over which they had been collected to identify those regions that could be examined more closely with a view to understanding temporal and spatial variations in the data. The number of samples varied from single data uploads on one date (North Carolina in the United State and Anping in Taiwan) to multiple samples over a prolonged period (155 samples in Koh Tao, over 5 years). The quality of time series analysis tends to improve with the length of the period of historical data being examined and the number of samples available, and so it was decided to further explore those regions that had collected a minimum of 50 samples and had a collection period of at least 3 years.

## Results

### Registration and participation analysis

An initial examination of the registration and participation data showed there were a total of 1035 separate registrations from 66 countries (Table S1, Supplementary Information). Great Britain had the highest number of registrations (*n* = 440), followed by the USA (*n* = 200) and Australia (*n* = 68). A total of 22 countries had only one registration and only 14 countries had more than 10 registrations. Data were also uploaded from six countries where the participants had registered from another country, bringing the total number of countries to 72. These included (1) samples from Antarctica, and the independent British Overseas Territories of the Falkland Islands and South Georgia (*n* = 9), all of which were submitted by passengers from a cruise ship organisation that registered for the project from Australia; (2) a sample from Oman submitted by a participant based in the USA; (3) samples submitted from Malta by a participant based in Great Britain. Of the total number of registrations, 111 were from low to middle-income countries. Sample data were also submitted by unregistered participants in Great Britain and Australia.

The total number of samples submitted was 1089 during the period of the study; this included 14 samples submitted from countries that had not been registered. Great Britain, Mozambique, and Thailand had the highest number of sample submissions. These data were attributed to 187 unique participant registration numbers, which was only 18% of the total number of participants that initially registered to take part in the project. Note that this figure was lower than the total number in Table S1 (*n* = 192) due to some participants submitting data from more than one country.

A closer analysis of registration and submission data for the 39 countries from where data were submitted (Table S2) showed that the country with the highest mean number of data submissions per total registrations was Mozambique (28.8), followed by Palau (19.0), whereas Great Britain and the USA, which had the highest number of registrations, were ranked 22nd and 28th, respectively. When the same analysis was undertaken based on the number of unique participants that submitted data in each country, Thailand had the highest mean (62.7), followed by Mozambique (38.3), and Great Britain and the USA ranked 12th and 18th, respectively.

Participants who registered or submitted data were grouped into seven organisational categories based on the information they provided (Table 1). By comparing the number of samples submitted by participants within each category, it was possible to determine a conversion rate that highlighted the level of participation within each of the organisational groups. Based on the total number of registrations, NGOs provided a higher number of surveys per registration than the other organisational groups. This figure increased by a factor of nearly five when based on the unique participants that submitted data. Individual participants proved to be the least effective group in terms of converting interest to actual results.

**Table 1:** Analysis of data by organisational affiliation

Organisational affiliation	Total registrations	Unique participants	Surveys submitted	Conversion based on total registrations %	Conversion based on unique participants %
Individuals	565	72	135	24	188
NGOs	195	49	753	386	1537
Universities	109	27	51	47	196
Companies	52	6	16	31	267
Schools	94	21	73	78	348
Government Agencies	20	7	43	215	614
Scouting Groups	9	1	4	44	400
**Totals**	**1035**	**183**	**1089**		

It was noticeable that there was a disproportionate spread of data submissions from NGOs. For example, although there were four registrations in total from Mozambique, all but one of the 115 data submissions were provided by two NGOs. Similarly, in Argentina, 54 of the submitted data were provided by one NGO, whilst in Malaysia, 62 of the submissions were provided by the same NGO, and only two other registered participants submitted data for that country. It was also noticeable that the registration data analysis could be skewed by the way that some participants who affiliated with organisations registered to participate; in Palau, the 19 data samples were submitted by five different schools all using the same registration number, whereas in Bahrain, the 31 sets of data were submitted by students from the university using 23 separate registration numbers.

### Global overview of sample data

A breakdown of total plastic count (*n* = 58,904) from data submitted from all 39 countries based on the characteristics and colours defined by the BMS protocol showed that the most common type of MP and MEP were pre-production pellets (nurdles), whilst the most prevalent colour was white (Tables S3 and S4). These data were averaged by dividing the plastic counts for each type by the total number of surveys undertaken, to highlight geographical variations (Tables S5 and S6).

**Fig. 1:**
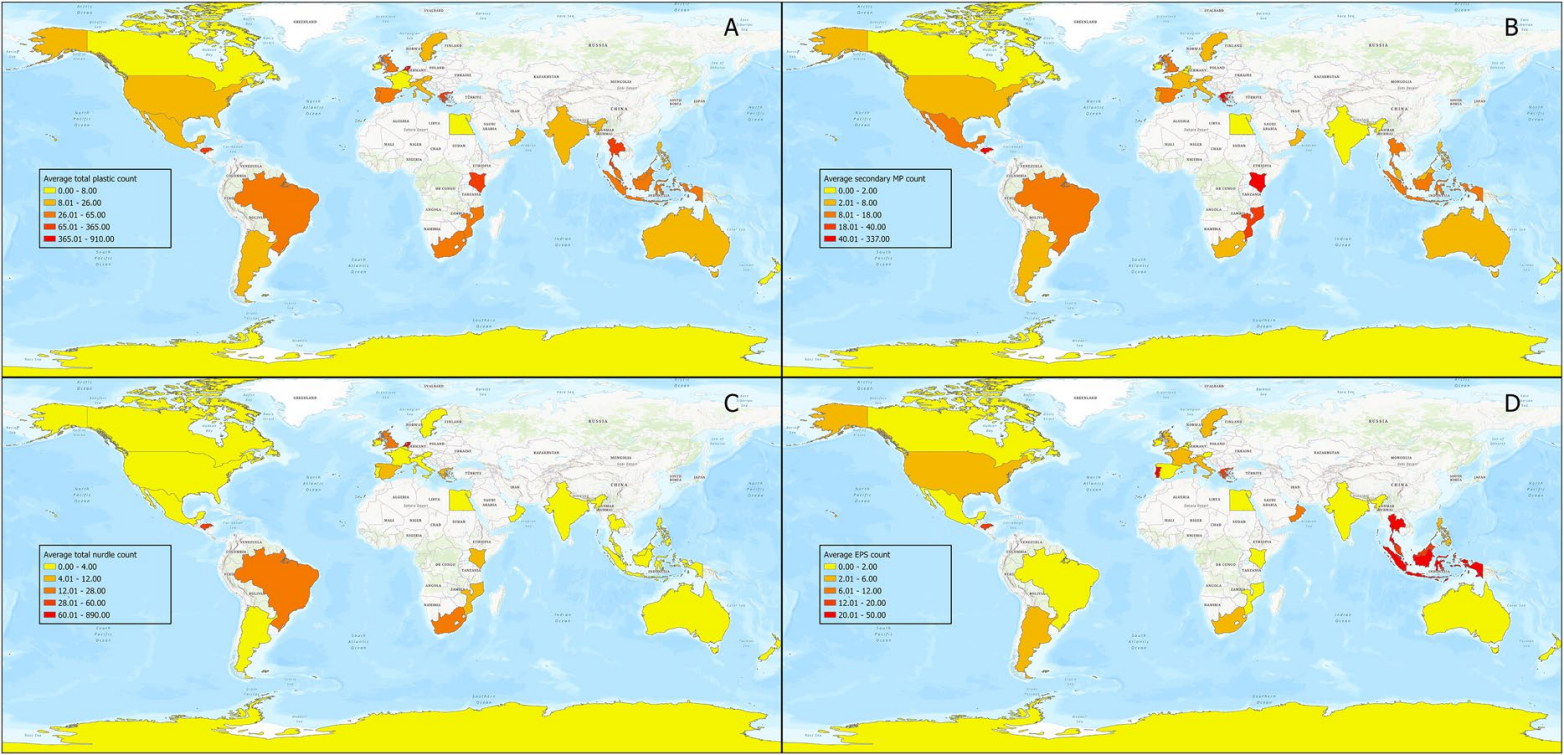
Average plastic distribution by type. **A** All plastic types, (**B**) Secondary MP, (**C**) Total Nurdles, and (**D**) Total EPS (Source: ESRI, Tom Tom, FAO, NOAA, USGS)

The countries with the highest average total counts were Netherlands, Honduras, Kenya, and Greece. The Netherlands had the highest average nurdle count, with more than 14 times the count of the next highest incidence, which was collected in Honduras; these accounted for much of the country’s total plastic count. The Netherlands also had the highest number of bio-beads. This contrasted with Kenya, where the plastic count mainly consisted of secondary MP. Honduras had the second highest average count of secondary MPs and the highest average count of secondary MEPs (81.0) which was nearly eight times higher than Cabo Verde (11.3). The average count of EPS balls was highest in Thailand, followed by Indonesia; however, Honduras had the highest incidence of EPS pieces, followed by Portugal. Cube nurdles and bio-beads were the least frequently encountered types of plastics. We visualised the distributions of the average counts of the most prevalent plastic types using ESRI mapping software (Fig. 1). Although the visualisation represents a global distribution, it was noted during the examination of the raw data that in many countries, there was localised bias, with samples being taken from a limited number of locations; for example, in Argentina, 88% of the samples were collected in the proximity of one coastal town, Puerto Madryn in Chubut Province.

**Fig. 2:**
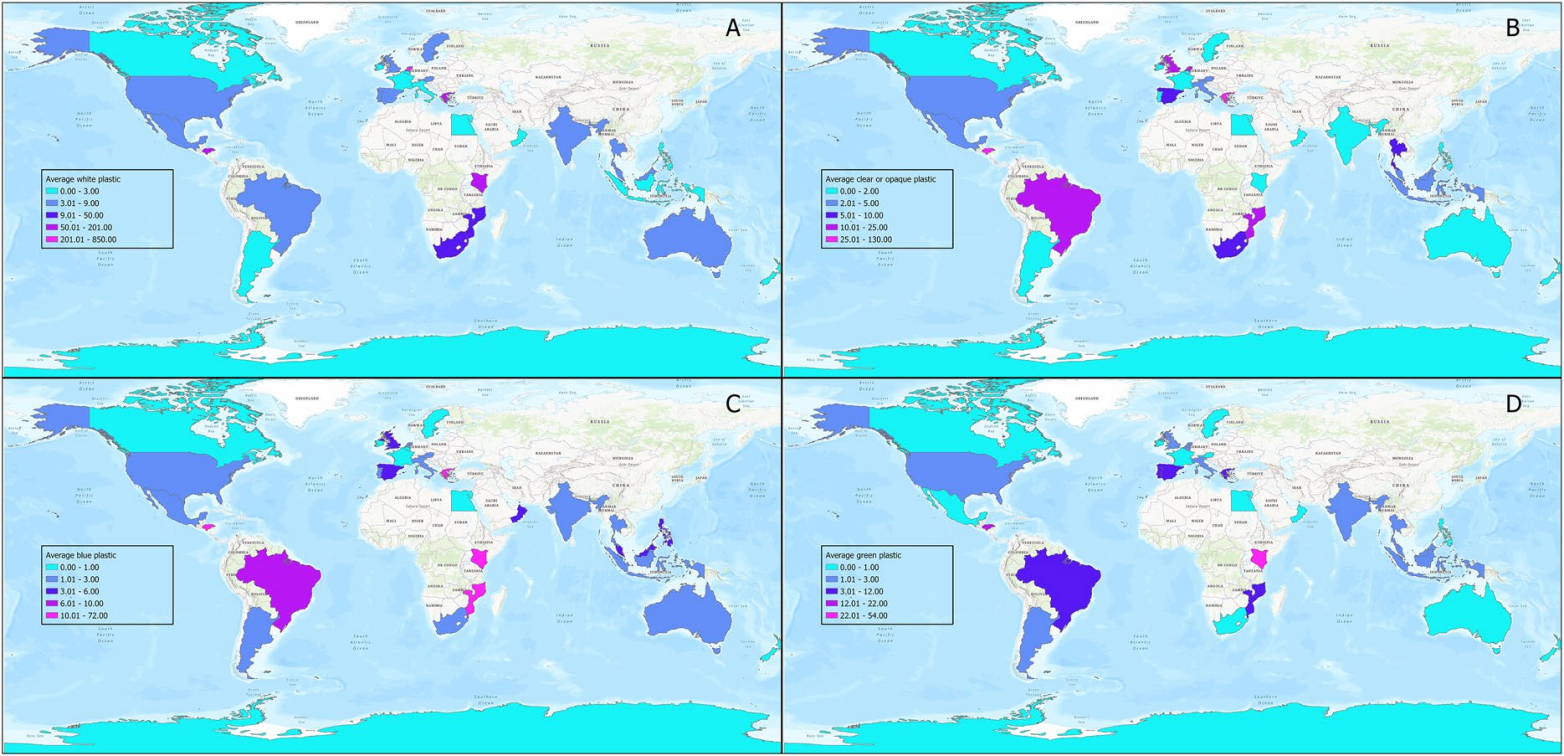
Average distributions of four most prevalent colours of plastic for each country. **A** White, (**B**) Clear or Opaque, (**C**) Blue, and (**D**) Green (Source: ESRI, Tom Tom, FAO, NOAA, USGS)

When colour data were analysed, white was the most prevalent colour, with nearly five times the normalised counts of the next most prevalent colour, which was clear or opaque, followed by blue and green. Note that all EPS was listed as white on the online data submission site for the BMS. Global distribution of the four most prevalent plastic colours was visualised using ESRI mapping software (Fig. 2).

### Regional analysis of sample data

Using the approach outlined in the methods section, we identified a total of 123 regions within the 39 countries (Table S7). The number of administrative regions within specific countries varied from single regions such as the Philippines and Ireland to those with multiple regions such as Great Britain and Palau. Regional data exploration was undertaken on countries that had data submissions from five or more regions; these were: Great Britain, Malaysia, Palau, and the USA.

**Fig. 3:**
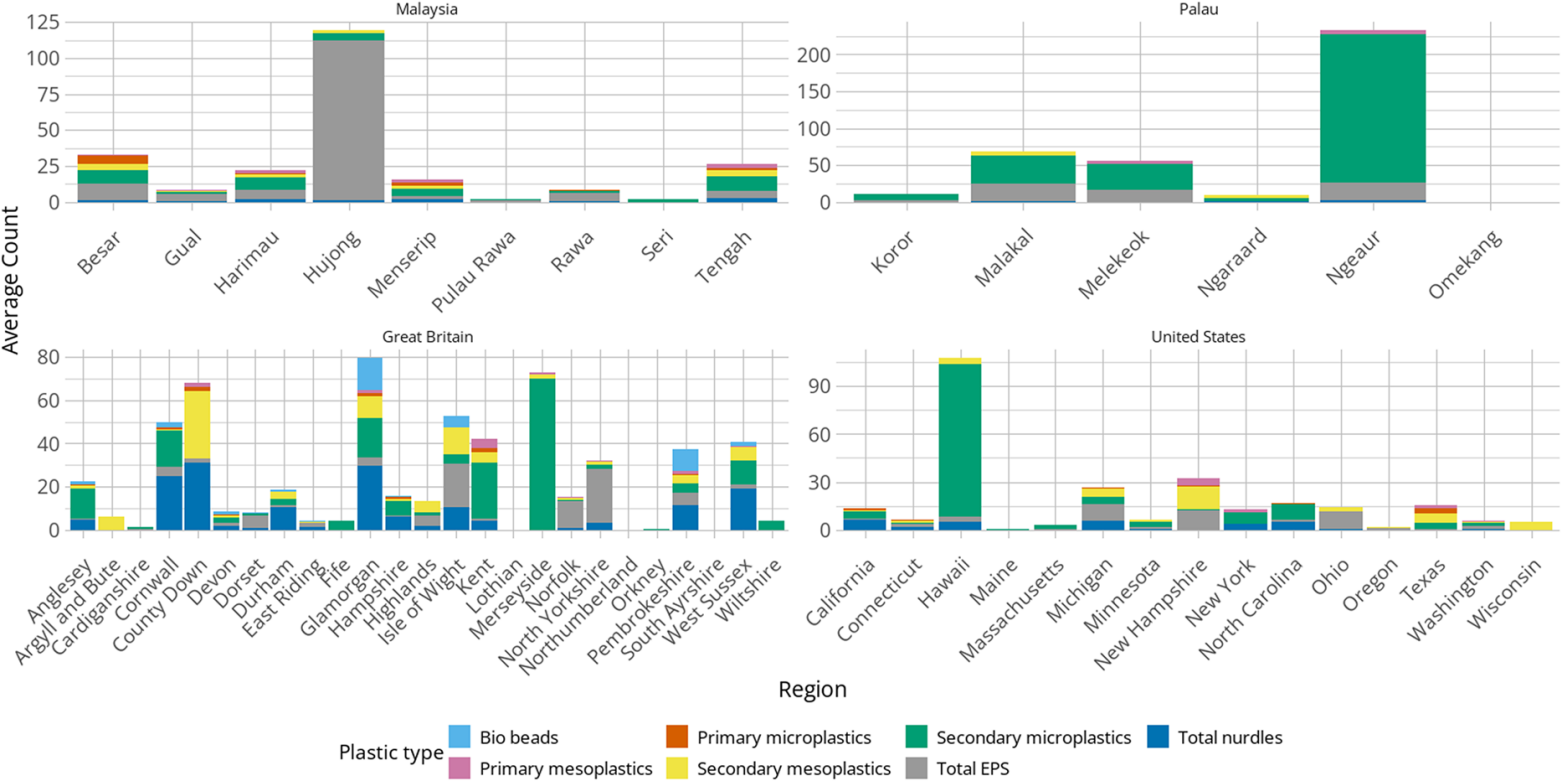
Regional analysis of plastic type for countries with five or more regions that submitted. Bar charts show average count per 0.005 m^3^ of sediment for each type of plastic

One of the variations noted in the global overview (Table S3) was the average counts of bio-beads found in different parts of the world, with the majority being found in the Netherlands and Honduras. Great Britain was ranked third in this category, and the regional analysis (Fig. 3) showed there was a noticeable difference in the incidence of bio-beads. In Great Britain, the total number of bio-beads reported was 542, and they were found in 11 of the regions; the highest concentrations were in Glamorgan (average count = 15) and Pembrokeshire (average count = 10) on the coast of Wales. This compared to only two in both the USA and Malaysia, and none in Palau.

Another feature that stood out from the Great Britain regional data was the high number of pre-production pellets (nurdles). The highest average count was found in County Down (*n* = 31.0) followed by Glamorgan (*n* = 29.8) and Cornwall (*n* = 24.8). It should be noted that only one survey was submitted from County Down, whilst four surveys were undertaken in Glamorgan and 30 in Cornwall. By contrast, nurdles were not as prevalent in the USA, but they were found in more than 50% of the regions surveyed. Nurdles were only found in small numbers in Malaysia and Palau, although they were present in all but two of the Malaysian islands surveyed.

Looking more closely at Palau, where 17 surveys were submitted, it was noticeable that much of the plastic pollution was secondary MP. These data were recorded in eight of the 17 survey sites. Of the total secondary MP found, 72.8% were located at one survey site to the south of the archipelago. Conversely, on the islands off the west coast of Malaysia, EPS was a noticeable pollutant, particularly in Pulau Hujong, where it accounted for 92.3% of the total plastic count. In the USA, bar chart one feature that stood out was the high average count of secondary MP found in Hawaii when compared to other US regions.

**Fig. 4:**
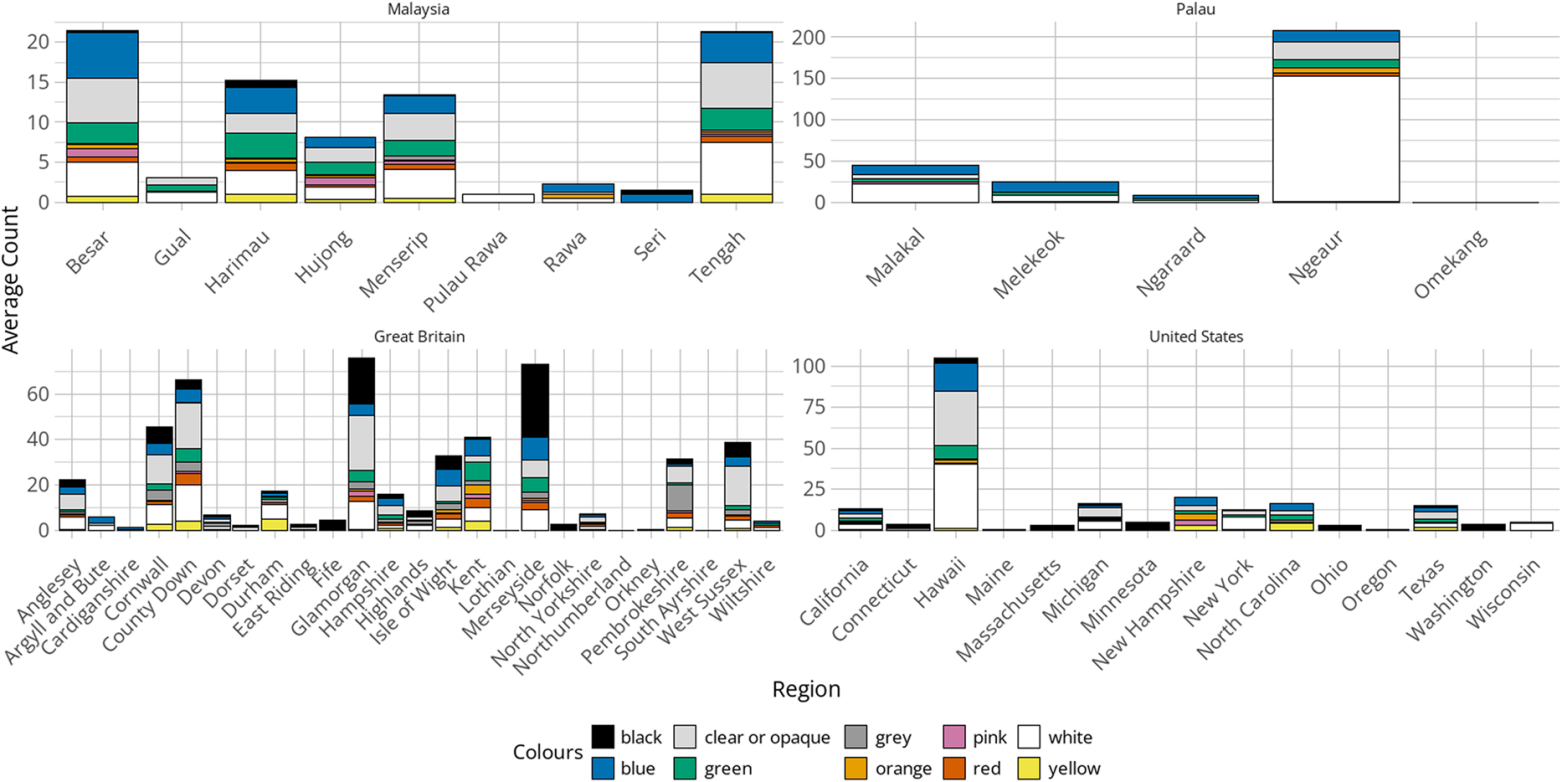
Regional analysis of plastic colour data for countries with five or more regions that had submitted data. Bar charts show average count per 0.005 m^3^ of sediment for each colour of plastic

An examination of the colour data of the samples from the same regions showed similar concentrations of clear or opaque, blue, green, and white plastics in the islands of Malaysia (Fig. 4). The islands of Palaua show less variation in colours, but a significant number of white plastics in Ngeaur (*n* = 305), whilst Great Britain had a spread of all the colours across counties. That said, there were higher amounts of black plastics in comparison to the other locations in Merseyside and Glamorgan. In the USA, there was a spread of colours in 50% of the regions surveyed, but there were limited amounts of black plastics.

### Local analysis of sample data

For a more local analysis based on the criteria outlined in the methods, we identified four regions that had more than 50 samples as well as more than three years of data collection (Table 2).

**Table 2:** Regions selected for final level of data exploration based on sampling period and number of samples submitted

Country	Region	First sample date	Last sample date	Total samples
TH	Koh Tao	17/06/2020	01/04/2024	155
MZ	Inhambane	25/10/2019	22/03/2024	60
MZ	Maputo	16/08/2018	04/03/2024	55
AR	Chubut	12/08/2019	29/02/2024	53

**Fig. 5:**
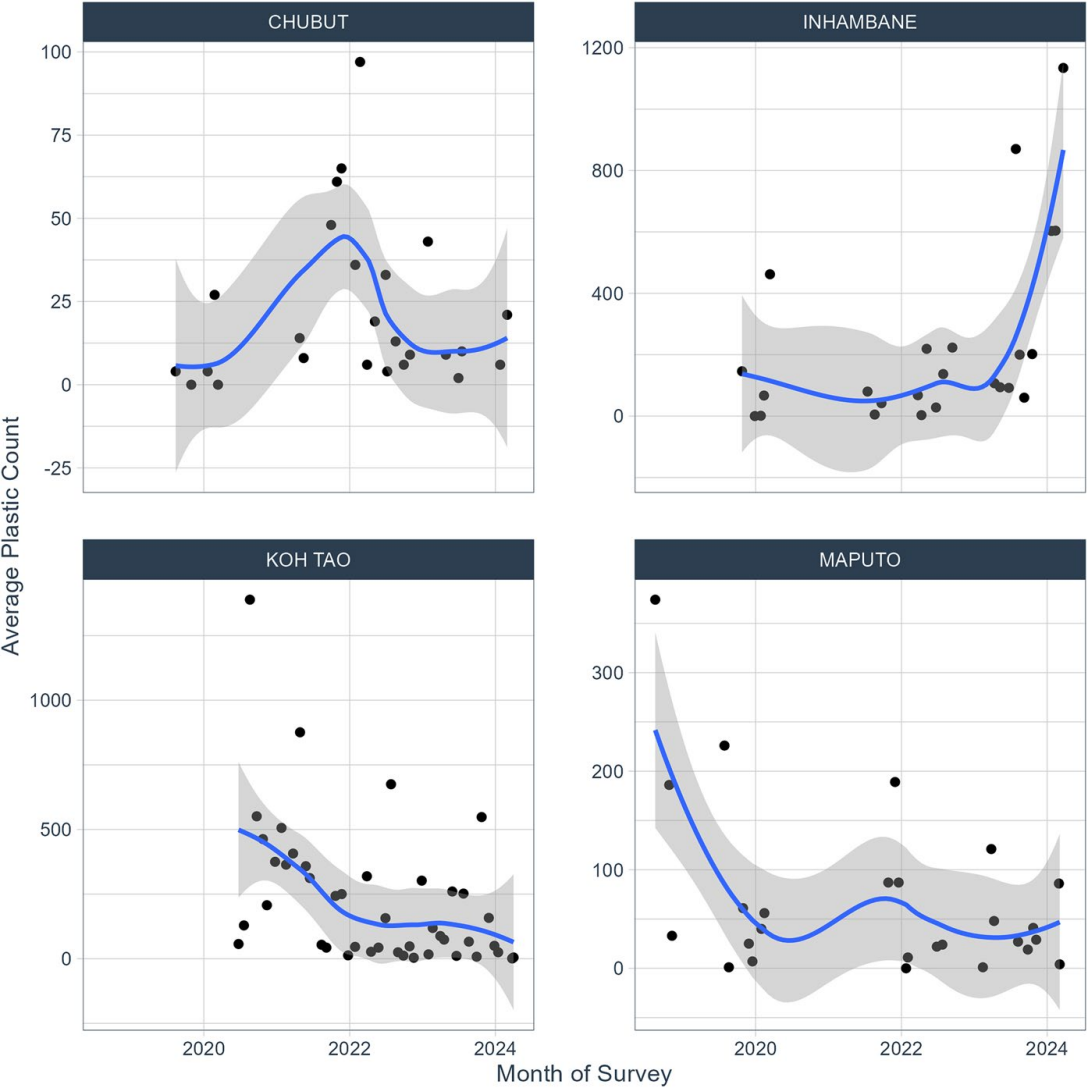
Time-series analysis of regional average total plastic count. A locally estimated scatterplot smoothing (LOESS) line has been added

Monthly data from each of these locations shows significant regional variations in average total plastic counts over time (Fig. 5).

**Fig. 6:**
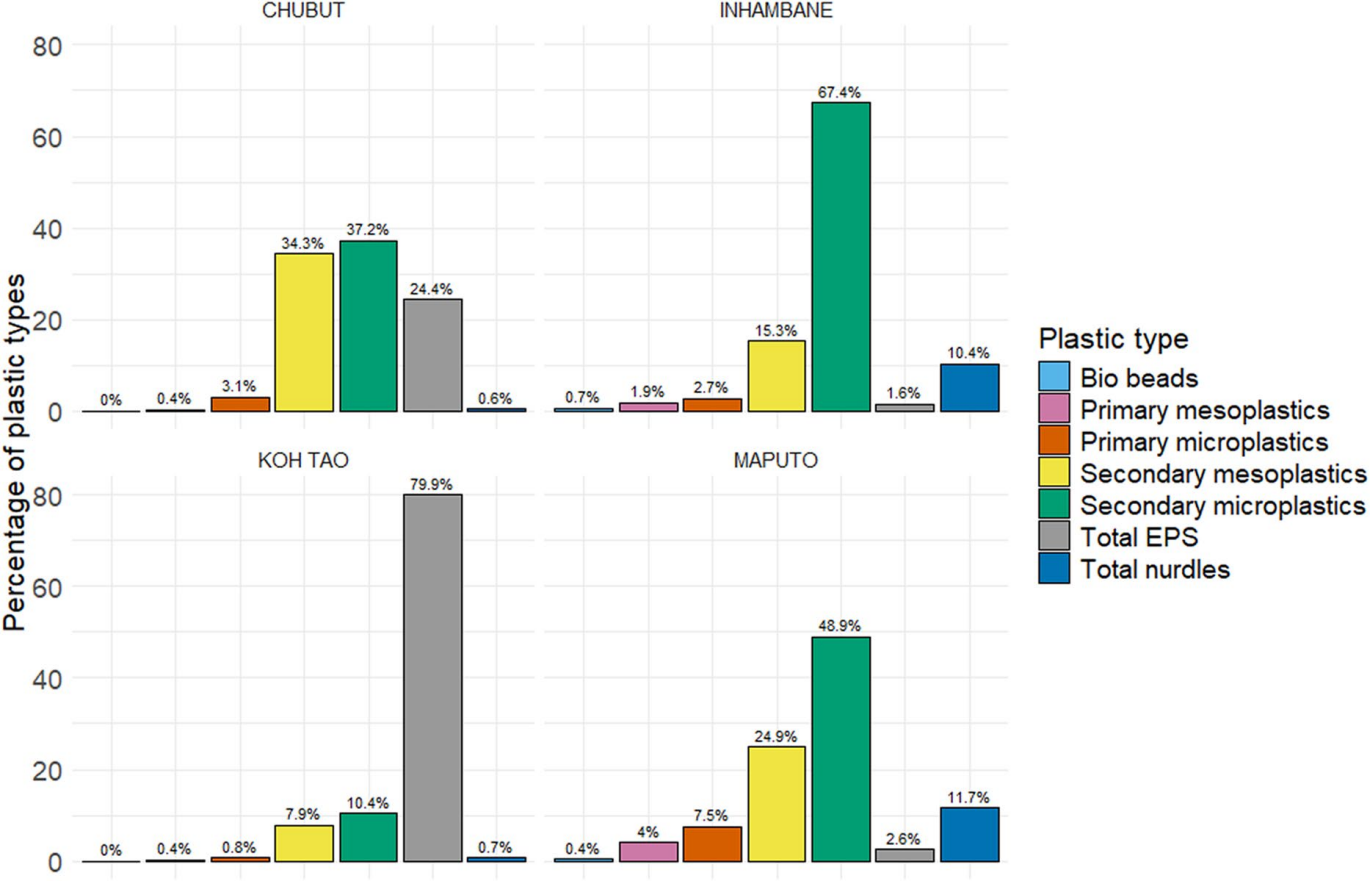
Percentage of types of plastics found in all four regions

As well as variations in total counts, the submitted data also highlighted variations in the proportions of types of plastics (Fig. 6). In Inhambane and Maputo, the percentage of secondary MP was noticeably higher than the other types of plastics found, whilst the proportion of nurdles found was similar in both locations. In Koh Tao, the number of EPS was a significant feature of the total count, whilst in Chubut, the proportion of secondary MPs and MEPs was similar.

**Fig. 7:**
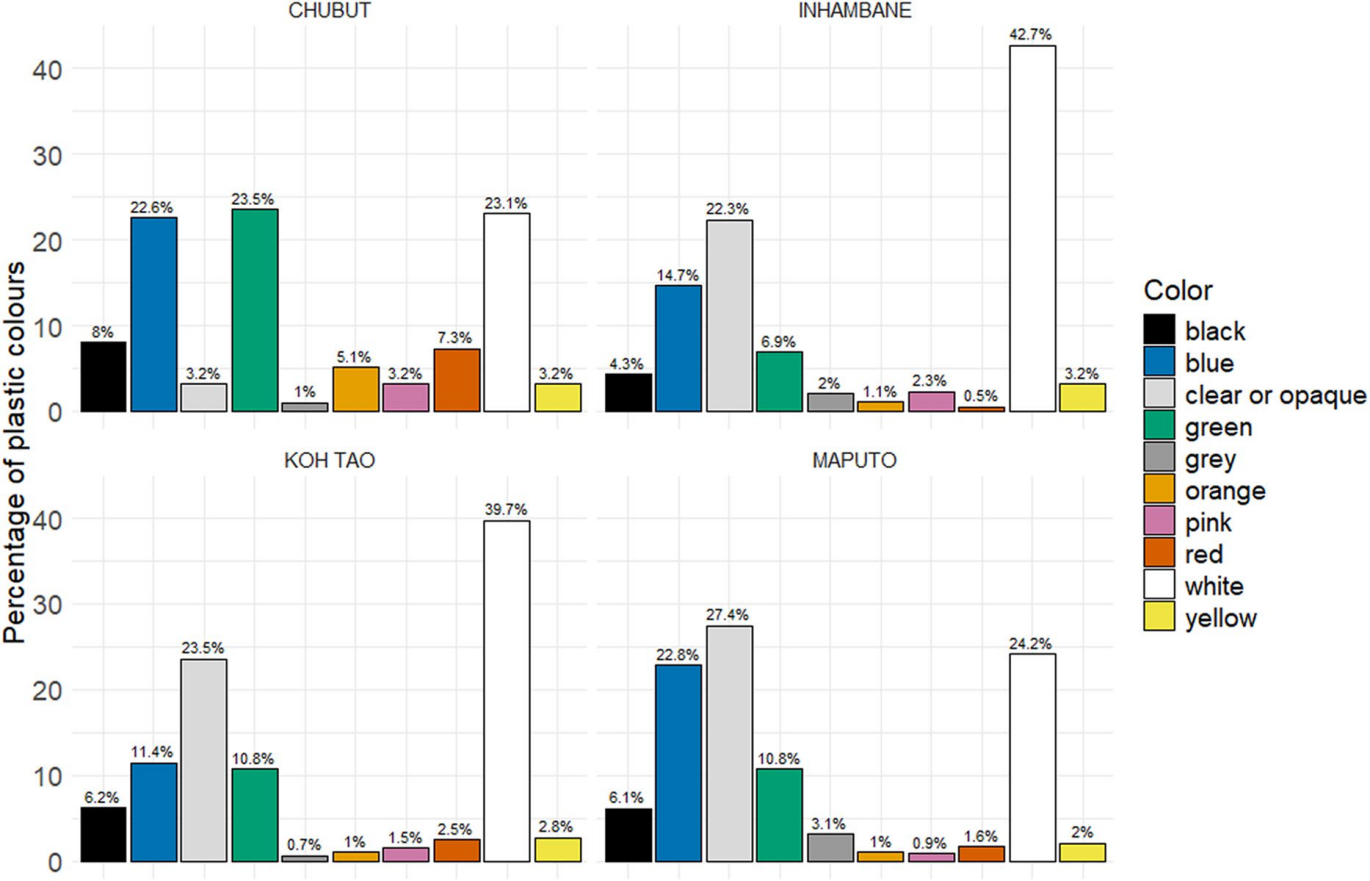
Percentage of colours of plastics found in all four regions

An examination of the variation of the colours of plastic data submitted showed some similarity between the distribution of colours in Inhambane and Koh Tao, but noticeably less white plastic in Maputo (Fig. 7). In Chubut, there were almost equal proportions of white, green, and blue plastics found.

**Fig. 8:**
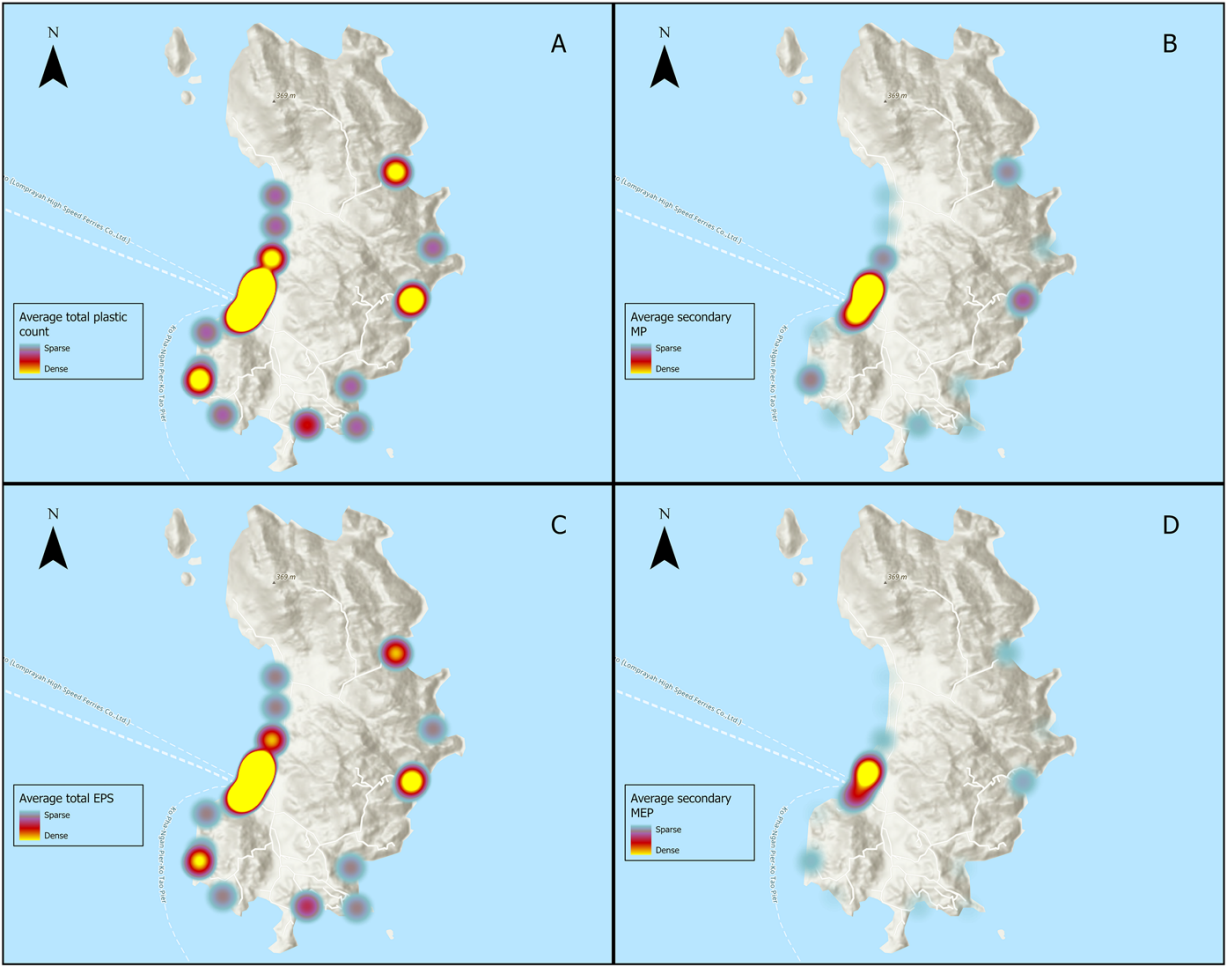
Multiple heat maps generated from sampled plastic data highlighting variations in plastic types around the island of Koh Tao. **A** Average total plastic count. **B** Average secondary MP. **C** Average total EPS. **D** Average secondary MEP (Source: ESRI, NASA, NGA, USGS, Tom Tom, FAO, NOAA)

Except for one sample, the data for the Maputo region of Mozambique were collected from the coastline around Punto do Ouro, approximately 125 km south of the country’s capital, Maputo. The data for the Inhambane region were collected from the Tofo Beach area, approximately 470 km north of Maputo. This limited geographical distribution of samples prevented any spatial analysis of the data. Similarly, an examination of the coordinates of the Chubut data showed that 47 out of the total of 53 surveys were undertaken in an area of coastline near the town of Puerto Madryn, and as a result, differentiating between spatial distribution offered limited results. In Koh Tao, however, the geographical spread of samples allowed for some spatial analysis, and heat maps were generated using ESRI mapping software to provide a visualisation of plastic distribution around the island based on average counts of total plastic compared with the distribution of the three most prevalent categories (Fig. 8).

## Discussion

Recent studies have demonstrated the important role that CS projects can play in managing plastic pollution in the marine and coastal environment by increasing our knowledge and understanding, educating and encouraging behavioural change, and potentially influencing policy (Dalby et al., 2021). The results of this study have demonstrated that the BMS project has the potential to provide similar benefits, and several advantages and challenges warrant further discussion.

### Registration and participation in CS projects

Only 18% of registrants to the BMS project submitted data (Tables S1 and S2) and this is a consistent challenge for CS initiatives where it can be difficult to maintain participant engagement from the initial interest stage through to making an actual contribution (Parrish et al., 2020; Pateman et al., 2021). Nielsen (2006) referred to this as the 90:9:1 percent rule, where a small percentage (1%) of volunteers contribute a significant proportion of data, whilst the majority provide only minor contributions (9%) or never contribute (90%) (Robinson et al., 2021). Whilst this may appear inefficient, high numbers of initial interest are necessary to achieve a critical mass of volunteers to enable the project to function (De Moor et al., 2019), and the engaged participants tend to submit consistent and high-quality data (Eveleigh et al., 2014; Jennett et al., 2016). Additionally, it should be remembered that the positive outcomes of CS projects are not just the data that the participants collect, but the additional benefits associated with participant engagement (Bonney et al., 2016; Phillips et al., 2015, 2019), such as increasing awareness and understanding of critical environmental issues. Whilst the situation is not ideal and more work needs to be done to better understand the barriers to full participation, it appears for now at least, that this pattern is the norm and realising this allows CS to continue to contribute positively to science and to the public in general (Lukyanenko et al., 2016; McKinley et al., 2017).

The primary focus of most CS initiatives remains on engaging and mobilising members of the public to volunteer and dedicate their free time to the projects (Dickinson et al., 2012; Phillips et al., 2019). However, the results clearly showed that organisations were far more productive than individuals when it came to submitting data (Table 1). Previous studies have shown that NGOs can address some of the barriers to participation often associated with CS participation by offering support, guidance, encouragement, and a sense of community (De Moor et al., 2019) and this study shows that the same argument can also be applied to schools, universities, government organisations, and other affiliated groups. Organisations can leverage their networks to attract volunteers and potentially enhance the quality and reach of CS programmes (Conrad & Hilchey, 2011; Shirk et al., 2012; Wiggins et al., 2013). So, whilst public engagement and the associated participatory benefits are important, given the often-limited resources available for long-term monitoring programmes, it could be argued that the focus of engagement for future research projects like the BMS should be to encourage organisational, rather than individual uptake. That said, a downside of this approach would be to reduce the opportunities to raise awareness and educate a wider audience. More research is needed in this area as well to ensure a balanced approach to CS project development that ensures efficient data collection, whilst meeting the wider benefit objectives.

### The value of a global overview

A strength of this study was the broad geographic coverage and extensive dataset provided by the BMS project. It allowed for a global overview that demonstrated the pervasive nature of MP and MEP pollution. With 1089 surveys submitted from 39 countries from diverse socio-economic backgrounds, the project revealed significant geographical variability in plastic pollution. It also served to highlight the widespread interest in the issue and the potential for CS initiatives to collect extensive environmental data, particularly in traditionally under-represented locations and low to middle-income countries. However, a limitation of the study was that although the total number of data samples was relatively high, they lacked sufficient statistical power to draw any definitive conclusions, or hypothesise broader trends from a country, regional, or local perspective (Vogel et al., 2013). This is a result of the opportunistic and unstructured nature of CS projects of this type when compared to more traditional scientific surveys. The issue was compounded by the zero-skewed nature of the data which complicated the application of traditional statistical models; for that reason, no inferential statistical analysis was undertaken. Despite the lack of inferential analysis, the descriptive analysis and data visualisation highlighted areas of interest and geographical patterns that are worthy of further investigation. For example, the high number of nurdles and bio-beads reported in the Netherlands stood out as anomalous (Fig. 1 and Table S3), and was clearly due to the MSC *Zoe* disaster in January 2019 (BBC, 2019), when large amounts of debris, including plastic nurdles and bio-beads, were spilled in the Wadden Sea (Hoogland, 2023; van der Molen et al., 2021). The accident significantly skewed the overall plastic pollution count for the country and influenced the distribution of colours of plastics (Fig. 2 and Table S4), as the majority of nurdles released were white, and bio-beads are predominantly black. This underscores the importance of contextualising data during analysis and considering any incidents that might have an abnormal impact on the data (Molina-Padrón et al., 2024). It also highlights the benefits that low-cost projects such as the BMS can offer in identifying the immediate distribution and impacts of environmental accidents.

Another example of geographical patterns highlighted by the study was the high level of secondary MP in Kenya, Greece, and Honduras. Whilst plastics are chemically engineered to be durable and resistant to degradation, under the right conditions they can become brittle and fragment into secondary MPs at a rate that varies depending on the physical processes they are subjected to, but which can take many years (Andrady, 2011; Barnes et al., 2009; Hidalgo-Ruz & Thiel, 2013; Roos Lundström & Mårtensson, 2015). The abundance and distribution of plastic debris are linked to a wide range of anthropogenic and natural coastal processes, and accumulation rates will vary (Browne et al., 2015; Cope & Wilkinson, 2014; Lee et al., 2015). Similarly, in Thailand and Malaysia, there were higher incidences of EPS than in other parts of the world, a feature that has been reported in previous research into EPOS pollution in Asia (Chan & Not, 2023). The identification of high numbers of types of plastic in specific locations through CS projects such as BMS not only supports existing research (Ita-Nagy et al., 2022; Kerubo et al., 2021; Tziourrou et al., 2019), it can also act as a catalyst for additional research; in this case, it would be worthwhile to examine these data alongside those anthropogenic and coastal processes. Linking these wider pollution patterns to urban settlements, human activities, and physical processes could potentially help towards the generation of management strategies for the mitigation of plastic pollution in those locations (Chan & Not, 2023; Lippiatt et al., 2013).

### The value of regional data

The regional and localised analyses allowed a more detailed examination of the data and again, whilst there were insufficient data to hypothesise specific causes for the coastal pollution, some features stood out. For example, the high incidence of bio-beads found in some Great Britain regions compared to the other countries was significant (Fig. 3). Bio-beads are released into the environment either due to mismanagement during transportation and logistical handling, or from being washed through damaged screens during the wastewater treatment process. A study undertaken by Turner et al. (2019) prior to the MSC *Zoe* incident reported on the distribution of bio-beads on 17 beaches in southern Great Britain and found that there was varying abundance, no distinct patterns, and only anecdotal evidence of bio-beads beyond Europe. Although sampling methods differed, our study supports those findings to some extent, with no specific patterns and a varied distribution (Figs. 3 and 4). A report by the Cornish Plastic Pollution Coalition (CPPC) (2018) showed the locations of water treatment plants using bio-beads as a filtration medium in southwest England, although a comparison of the locations of those sites and the occurrence of bio-beads in our surveys did not reveal any correlation. What is unknown is why so few bio-beads were encountered in other locations, particularly regions in the USA. According to the CPPC report, the USA has recorded significant losses of bio-beads into the environment, yet only two pieces were reported during the surveys.

The regional analysis also showed a relatively high incidence of nurdles in regions in Great Britain in comparison to the other countries, particularly on the coastlines of Glamorgan and County Down (Fig. 3). Like bio-beads, nurdles enter the environment through accidental loss during use or mishandling during transportation (Ogata et al., 2009) and their presence is often associated with industrialised areas; however, the sample sites in Glamorgan and County Down were in rural areas, suggesting that they had been brought to that location by physical processes, ocean currents. In Hawaii, the significant presence of secondary MPs might have been expected due to its proximity to the centre of the North Pacific Gyre, a known location where marine debris concentrates due to circulatory ocean currents (Chen et al., 2018). Additionally, the data analysed in this research show similarity to the results found in previous studies in Hawaii; Young and Elliott (2016) found that 93.2% of the plastics collected from the same two islands in Hawaii sampled by BMS volunteers were secondary fragments of larger plastic debris. This compares with the 95.3% of secondary MPs found in Hawaii through BMS. With regard to the colours of plastics, Young and Elliott also found the most common colour was white (71.8%) followed by blue (8.5%), green (7.5%), and black (7.3%). This compares with the most common colours found by BMS volunteers of white (including clear and opaque) (65.1%), blue (15.1%), green (7.2%), and black (3.0%). Palau, which is also on the edge of the North Pacific Gyre, had similarly high proportions of secondary MPs and only small numbers of bio-beads and nurdles. The highest incidence of plastics was recorded in Ngeaur (total count *n* = 466) and this was predominantly secondary MPs (86.1%) followed by EPS (10.7%). This prevalence beyond the more urbanised parts of the archipelago supports the findings of Béraud et al. (2022) during their Palau research and the significance of ocean currents rather than human activity in plastic pollution distribution. In Malaysia, a significant finding was the high incidence of EPS, which accounted for 50.3% of the total plastic reported. This elevated level of EPS contamination supports previous research into the prevalence of EPS on Asian coastlines, which shows that 40.4% of plastic found is EPS in comparison to 17.3% in the rest of the world (Chan & Not, 2023). The light weight of EPS means that it is readily transported by several natural coastal and marine processes, and long-term monitoring programmes will be essential to increasing our understanding of its distribution dynamics.

Whilst it is not possible to make any statistical inference from these data, all three examples provide supporting evidence in our understanding of the sources and pathways of MP and MEP pollution and the impact of physical factors on spatio-temporal variability. This information is essential for devising regional management strategies and targeted interventions (Balthazar-Silva et al., 2020; Frère et al., 2017; Launio & Pimentel, 2024).

### The value of local data

The localised analysis provided more detail about coastal MP and MEP distribution dynamics in four specific locations. A time-series analysis (Fig. 5) provided a limited visual representation of the data in four regions, but for a more detailed analysis of trends, consistent and repeated measurements over time are required. In this study, the sporadic and irregular nature of sample collection, coupled with insufficient data from the specific regions, made it difficult to draw conclusions, particularly given the high number of zero counts (Ducklow et al., 2009). In fact, the LOESS smoothing line appears to be influenced by outliers in all four regions, which is indicative of the problem of insufficient samples. That said, this study does show the potential of the BMS to capture the complexities of plastic pollution trends over time, especially in those areas that might otherwise be overlooked and under-represented by traditional scientific research. A concentrated effort, working in collaboration with NGOs and providing them with additional resources where necessary, could potentially provide more meaningful results in the future.

From the analysis of localised types and colours of plastics, it was noticeable that none of the regions follow the plastic type patterns found during the global overview of the data, where the dominant plastic types were nurdles (Fig. 6). In the two sample locations in Mozambique, the proportions of the various types of plastic were different, although the ranking of each type was the same. Given the distance between the two locations, it is a feature worthy of further investigation that might fill some of the existing gaps in our knowledge of the region and the impact of the Indian Ocean Gyre on plastic pollution distribution (Connan et al., 2021). Similarly, the analysis of colour shows that only Koh Tao and Inhambane followed the colour patterns found during the global overview (Fig. 7). In both Chubut and Maputo, the distribution of colours found was dissimilar to the global overview of the data. In Koh Tao, the proportion of colour has been compounded by type, and the high percentage of white can be linked to the high prevalence of EPS, which can only be submitted as white on the BMS upload webpage. Why these four regions varied so much from the global distribution patterns of plastic types and colours is uncertain; however, it does show to some extent the impact of local variation and the potential impact of coastal communities. Whilst the distribution of sample sites prevented any spatial analysis from being undertaken in three of the regions, it was possible in Koh Tao. The heat maps (Fig. 8) clearly identified those locations where plastic concentrations were highest, and they also highlight the relationship between total plastic count and total EPS, which supports the work of Chan and Not (2023). Most of the plastics and EPS in Koh Tao were found around Mae Haad on the west of the island, where the main town, a ferry pier, a small dock, and numerous hotels and businesses are located, highlighting possible links to anthropogenic activity. However, three other plastics ‘hotspots’ were also identified in small bays, two on the east coast and one in the southwest, highlighting the importance of physical coastal processes in distribution dynamics. This type of specific localised data can play an important role in identifying sources and pathways, engaging local communities, developing community-based management approaches, empowering local stakeholders to act, and encouraging behavioural change (Dalby et al., 2021; Henderson & Green, 2020).

### Summary of analysis and recommendations

The aim of this study was to understand better how CS research can be used to study coastal MP and MEP distributions. The registration process used by the BMS project was not without issues; the disparity between interest and engagement in the registration process needs to be addressed, and more detailed instructions need to be included to prevent multiple registrations by organisations. However, the study served to emphasise how organisational collaboration can benefit long-term monitoring programmes and provide an opportunity to collect more data than might otherwise be possible. During the global analysis, we identified errors in the submission of sample coordinates, and there were clear incidents of spatial and temporal bias. The first issue can be addressed by recent advances in technology, particularly in mobile applications, improving the accuracy of data collection by providing real-time location tracking and standardised data entry formats (Hu et al., 2019). The second issue is less easily resolved; by its very nature, CS tends to be opportunistic, and real-world constraints limit the options of volunteers. Kays et al. (2021) recommended a hybrid approach, combining traditional and CS science by supplementing CS efforts with professional data collection in areas where results were limited. Whilst we support this view where it is feasible, we do not believe that this should be at the expense of limiting what CS can collect, especially given the additional benefits to individuals and coastal communities (Anderson & Wentworth, 2014; Cigliano et al., 2015). Moreover, studies have shown that spatial and temporal bias in CS data can be effectively managed through careful project design and data analysis, along with statistical methods to account for variability in the sampling effort (Mandeville et al., 2022). We believe the emphasis should be on working with the data provided through analytical and process techniques rather than limiting the enthusiasm of citizen scientists and ignoring the opportunity they provide to the scientific community.

The purpose of this study was not intended to prove definitively the cause or source of any type or colour of MP and MEP pollution. Instead, it was designed to examine the application of CS methods to determine whether they might support more traditional scientific data collection methods. The analysis of these data has provided an opportunity to identify structural issues in the process of data collection and anomalies and patterns within the data that can serve as a catalyst for further research. When coupled with existing and future research, CS research can help to provide a better understanding of the coastal plastic pollution issues, which is a crucial element for further intervention and the development of effective mitigation strategies, policies, and local community initiatives (Jambeck et al., 2015). We believe this study has demonstrated that CS data can make a valuable contribution to our understanding of the distribution of coastal MPs and MEPs in support of traditional scientific methods and potentially play a role in the long-term monitoring of coastal plastic pollution.

## Conclusions

Plastic pollution is a global issue with a widespread distribution, affecting even the most remote and pristine environments. Traditional scientific methods for monitoring plastic pollution are often limited by high costs, logistical challenges, and the inability to cover vast and diverse geographical areas. At the same time, long-term monitoring programmes often face the challenge of limited resources. Citizen science projects like the BMS provide a cost-effective and scalable solution to these challenges by harnessing the power of the public to collect data across different regions and time periods. This research highlighted that even with a limited amount of data, it was possible to identify anomalies and patterns that can provide the basis either for further research or for direct action that can not only have an impact on local communities but also play a role in supporting international agreements. This study has shown that although there are aspects of this CS project that could be improved, CS data has an important role to play in supporting traditional science in the study of coastal MP and MEP distribution, and in helping to engage with stakeholders at all levels, including local communities, in their efforts towards finding solutions.

## Supplementary Information

Below is the link to the electronic supplementary material.

Supplementary file1 (DOCX 74.5 KB)

## Data Availability

No datasets were generated or analysed during the current study.

## Footnotes

1 Bio-beads are used in the treatment of wastewater. They are similar in size to pre-production plastic pellets (nurdles), normally black, and have an irregular shape and a rough, or dimpled surface (Turner et al., 2019).

## Declarations

All authors have read, understood, and have complied as applicable with the statement on ‘Ethical responsibilities of Authors’ as found in the Instructions for Authors.
