# Supplementary material for: The potential contribution of citizen science data in the study of coastal microplastic and mesoplastic distributions
Source: Environ Monit Assess. 2025 Jul 21;197(8):936. doi: 10.1007/s10661-025-14354-2 (PMC12279598; doi:10.1007/s10661-025-14354-2)
Supplement: Supplementary file 1 — Supplementary file1 (DOCX 74.5 KB) [file 10661_2025_14354_MOESM1_ESM.docx]

Supplementary Information - Tables

Table S1. Global registration and submission data submitted to the BMS project between March 2018 to April 2024.

| **Serial number** | **ISO Alpha-2 country code** | **Country of registration** | **Total number of registrations** | **Number of registrations that submitted data** | **Total number of samples submitted** |
| --- | --- | --- | --- | --- | --- |
| 1 | AE | UAE | 2 | 0 | 0 |
| 2 | AQ | Antarctica | 0 | 2^(a)^ | 7 |
| 3 | AR | Argentina* | 4 | 2 | 56 |
| 4 | AT | Austria | 1 | 1 | 1 |
| 6 | AU | Australia | 68 | 11^(a)(b)^ | 13 |
| 7 | BD | Bangladesh* | 1 | 0 | 0 |
| 8 | BE | Belgium | 1 | 1 | 2 |
| 9 | BG | Bulgaria | 1 | 0 | 0 |
| 10 | BH | Bahrain | 37 | 23 | 31 |
| 11 | BR | Brazil* | 3 | 2 | 3 |
| 12 | BS | Bahamas | 1 | 0 | 0 |
| 13 | BZ | Belize* | 1 | 0 | 0 |
| 14 | CA | Canada | 32 | 4 | 6 |
| 15 | CH | Switzerland | 4 | 0 | 0 |
| 16 | CL | Chile | 1 | 0 | 0 |
| 17 | CN | China | 2 | 0 | 0 |
| 18 | CV | Cabo Verde* | 1 | 1 | 3 |
| 19 | DE | Germany | 5 | 0 | 0 |
| 20 | DZ | Algeria* | 2 | 0 | 0 |
| 21 | EC | Ecuador* | 1 | 0 | 0 |
| 22 | EE | Estonia | 1 | 0 | 0 |
| 23 | EG | Egypt* | 3 | 1 | 3 |
| 24 | ES | Spain | 14 | 4 | 6 |
| 25 | FJ | Fiji* | 1 | 0 | 0 |
| 26 | FK | Falkland Islands | 0 | 1^(a)^ | 1 |
| 27 | FR | France | 7 | 3 | 3 |
| 28 | GB | Great Britain | 440 | 55^(b)(c)^ | 333 |
| 29 | GI | Gibraltar | 1 | 0 | 0 |
| 30 | GR | Greece | 3 | 2 | 2 |
| 31 | GS | South Georgia | 0 | 1^(a)^ | 1 |
| 32 | HK | Hong Kong | 2 | 2 | 4 |
| 33 | HN | Honduras* | 2 | 1 | 2 |
| 34 | HR | Croatia | 1 | 0 | 0 |
| 35 | ID | Indonesia* | 4 | 2^(c)^ | 14 |
| 36 | IE | Ireland | 11 | 1 | 1 |
| 37 | IN | India* | 18 | 3 | 16 |
| 38 | IS | Iceland | 1 | 0 | 0 |
| 39 | IT | Italy | 8 | 2 | 17 |
| 40 | KE | Kenya* | 2 | 1 | 1 |
| 41 | KR | South Korea | 2 | 0 | 0 |
| 42 | KY | Cayman Islands | 2 | 0 | 0 |
| 43 | LK | Sri Lanka* | 3 | 0 | 0 |
| 44 | LT | Lithuania | 1 | 0 | 0 |
| 45 | MT | Malta | 0 | 1^(c)^ | 2 |
| 46 | MV | Maldives* | 1 | 0 | 0 |
| 47 | MX | Mexico* | 12 | 3^(c)^ | 38 |
| 48 | MY | Malaysia* | 13 | 3 | 68 |
| 49 | MZ | Mozambique* | 4 | 3 | 115 |
| 50 | NG | Nigeria* | 1 | 0 | 0 |
| 51 | NL | Netherlands | 32 | 3 | 22 |
| 52 | NO | Norway | 4 | 0 | 0 |
| 53 | NZ | New Zealand | 9 | 2 | 10 |
| 54 | OM | Oman | 0 | 1 | 1 |
| 55 | PE | Peru* | 2 | 0 | 0 |
| 56 | PF | French Polynesia | 0 | 2^(d)^ | 2 |
| 57 | PH | Philippines* | 2 | 1^(c)^ | 2 |
| 58 | PR | Puerto Rico | 4 | 0 | 0 |
| 59 | PT | Portugal | 14 | 3 | 4 |
| 60 | PW | Palau | 1 | 1 | 19 |
| 61 | SA | Saudi Arabia | 1 | 0 | 0 |
| 62 | SC | Seychelles | 2 | 0 | 0 |
| 63 | SE | Sweden | 2 | 1 | 2 |
| 64 | SH | Saint Helena* | 2 | 0 | 0 |
| 65 | TH | Thailand* | 12 | 3 | 188 |
| 66 | TR | Turkey* | 1 | 0 | 0 |
| 67 | TT | Trinidad & Tobago | 1 | 0 | 0 |
| 68 | TW | Taiwan | 3 | 2^(d)^ | 5 |
| 69 | TZ | Tanzania* | 1 | 0 | 0 |
| 70 | US | United States of America | 200 | 34^(c)^ | 80 |
| 71 | UY | Uruguay | 2 | 0 | 0 |
| 72 | ZA | South Africa* | 14 | 3 | 5 |
|  |  | **Totals** | **1035** | **192** | **1089** |

Notes: *Denotes low and middle-income countries based on Organisation for Economic Co-operation and Development (OECD) statistics (OECD, 2024). (a) One registered volunteer submitted date from four countries. (b) One data sample was submitted by an unregistered participant. (c) One registered volunteer submitted data collected in six countries. (d) One registered volunteer submitted data from two countries.

Table S2. Data analysis based on unique registrations that submitted data to the BMS project during the period March 2018 to April 2024.

| **Serial number** | **ISO Alpha-2 country code** | **Country** | **Number of unique registrations that submitted data** | **Mean number of data submissions based on total registrations** | **Mean number of data submissions based on unique registrations that submitted data** |
| --- | --- | --- | --- | --- | --- |
| 1 | AQ | Antarctica | 1 |  | 7.0 |
| 2 | AR | Argentina | 2 | 14.0 | 28.0 |
| 3 | AT | Austria | 1 | 1.0 | 1.0 |
| 4 | AU | Australia | 10 | 0.2 | 1.3 |
| 5 | BE | Belgium | 1 | 2.0 | 2.0 |
| 6 | BH | Bahrain | 23 | 0.8 | 1.3 |
| 7 | BR | Brazil | 2 | 1.0 | 1.5 |
| 8 | CA | Canada | 3 | 0.2 | 2.0 |
| 9 | CV | Cabo Verde | 1 | 3.0 | 3.0 |
| 10 | EG | Egypt | 1 | 1.0 | 3.0 |
| 11 | ES | Spain | 4 | 0.4 | 1.5 |
| 12 | FK | Falkland Islands | 1 |  | 1.0 |
| 13 | FR | France | 3 | 0.4 | 1.0 |
| 14 | GB | Great Britain | 55 | 0.8 | 6.1 |
| 15 | GR | Greece | 2 | 0.7 | 1.0 |
| 16 | GS | South Georgia | 1 | N/A | 1.0 |
| 17 | HK | Hong Kong | 2 | 2.0 | 2.0 |
| 18 | HN | Honduras | 1 | 1.0 | 2.0 |
| 19 | ID | Indonesia | 2 | 3.5 | 7.0 |
| 20 | IE | Ireland | 1 | 0.1 | 1.0 |
| 21 | IN | India | 2 | 0.9 | 8.0 |
| 22 | IT | Italy | 2 | 2.1 | 8.5 |
| 23 | KE | Kenya | 1 | 0.5 | 1.0 |
| 24 | MT | Malta | 1 |  | 2.0 |
| 25 | MX | Mexico | 3 | 3.2 | 12.7 |
| 26 | MY | Malaysia | 3 | 5.2 | 22.7 |
| 27 | MZ | Mozambique | 3 | 28.8 | 38.3 |
| 28 | NL | Netherlands | 3 | 0.7 | 7.3 |
| 29 | NZ | New Zealand | 2 | 1.1 | 5.0 |
| 30 | OM | Oman | 1 |  | 1.0 |
| 31 | PF | French Polynesia | 2 |  | 1.0 |
| 32 | PH | Philippines | 1 | 1.0 | 2.0 |
| 33 | PT | Portugal | 3 | 0.3 | 1.3 |
| 34 | PW | Palau | 1 | 19.0 | 19.0 |
| 35 | SE | Sweden | 1 | 1.0 | 2.0 |
| 36 | TH | Thailand | 3 | 15.7 | 62.7 |
| 37 | TW | Taiwan | 2 | 1.7 | 2.5 |
| 38 | US | United States | 34 | 0.4 | 2.4 |
| 39 | ZA | South Africa | 2 | 0.4 | 2.5 |
|  |  | **Totals** | **187** | **3.5** | **6.6** |

Note: Those countries that did not have any registrations are not included in the column showing mean data submissions based on initial registrations.

Table S3. Summary of total plastic counts based on types by country.

| **Country ISO Alpha-2 code** | **Country** | **Nurdle spherical** | **Nurdle cylindrical** | **Nurdle disk** | **Nurdle cube** | **Bio beads** | **Other primary MP** | **Secondary MP** | **Primary MEP** | **Secondary MEP** | **Expanded polystyrene (EPS) balls** | **Expanded polystyrene (EPS) pieces** |
| --- | --- | --- | --- | --- | --- | --- | --- | --- | --- | --- | --- | --- |
| AQ | Antarctica | 0 | 0 | 0 | 0 | 0 | 0 | 1 | 0 | 0 | 0 | 0 |
| AR | Argentina | 0 | 3 | 0 | 0 | 0 | 17 | 207 | 3 | 194 | 91 | 42 |
| AT | Austria | 0 | 0 | 0 | 0 | 0 | 0 | 5 | 3 | 7 | 0 | 1 |
| AU | Australia | 10 | 18 | 14 | 3 | 3 | 3 | 46 | 4 | 28 | 6 | 0 |
| BE | Belgium | 0 | 0 | 0 | 0 | 0 | 3 | 1 | 1 | 0 | 0 | 0 |
| BH | Bahrain | 2 | 9 | 0 | 0 | 9 | 28 | 45 | 23 | 96 | 5 | 6 |
| BR | Razil | 47 | 0 | 10 | 0 | 2 | 32 | 54 | 8 | 3 | 1 | 6 |
| CA | Canada | 0 | 0 | 0 | 0 | 1 | 4 | 5 | 0 | 0 | 0 | 1 |
| CV | Cabo Verde | 2 | 0 | 0 | 0 | 0 | 0 | 14 | 1 | 34 | 0 | 0 |
| EG | Egypt | 1 | 0 | 0 | 0 | 0 | 0 | 4 | 0 | 1 | 0 | 0 |
| ES | Spain | 7 | 12 | 8 | 4 | 0 | 16 | 72 | 3 | 33 | 0 | 3 |
| FK | Falkland Islands | 0 | 0 | 0 | 0 | 0 | 0 | 0 | 0 | 0 | 0 | 0 |
| FR | France | 1 | 2 | 1 | 0 | 0 | 1 | 7 | 0 | 0 | 12 | 0 |
| GB | Great Britain | 558 | 1394 | 1986 | 311 | 542 | 106 | 2743 | 88 | 1250 | 736 | 796 |
| GR | Greece | 1 | 4 | 14 | 0 | 0 | 0 | 389 | 0 | 9 | 7 | 18 |
| GS | South Georgia | 0 | 0 | 0 | 0 | 0 | 0 | 0 | 0 | 0 | 0 | 0 |
| HK | Hong Kong | 73 | 1 | 0 | 2 | 3 | 0 | 6 | 5 | 2 | 33 | 26 |
| HN | Honduras | 70 | 34 | 7 | 9 | 7 | 2 | 398 | 0 | 162 | 3 | 37 |
| ID | Indonesia | 5 | 2 | 0 | 0 | 0 | 0 | 152 | 4 | 18 | 343 | 154 |
| IE | Ireland | 0 | 0 | 0 | 0 | 0 | 0 | 0 | 0 | 0 | 0 | 0 |
| IN | India | 34 | 0 | 0 | 0 | 2 | 27 | 29 | 11 | 64 | 1 | 6 |
| IT | Italy | 0 | 31 | 8 | 0 | 0 | 2 | 104 | 7 | 52 | 18 | 53 |
| KE | Kenya | 1 | 9 | 0 | 0 | 0 | 3 | 337 | 0 | 0 | 0 | 0 |
| MT | Malta | 0 | 0 | 0 | 0 | 0 | 1 | 14 | 0 | 0 | 12 | 3 |
| MX | Mexico | 0 | 0 | 0 | 0 | 0 | 3 | 321 | 16 | 97 | 3 | 6 |
| MY | Malaysia | 60 | 25 | 10 | 2 | 2 | 151 | 488 | 88 | 213 | 653 | 399 |
| MZ | Mozambique | 326 | 176 | 117 | 157 | 46 | 284 | 4554 | 178 | 1282 | 20 | 112 |
| NL | Netherlands | 6033 | 37 | 12744 | 13 | 821 | 6 | 7 | 149 | 7 | 0 | 1 |
| NZ | New Zealand | 3 | 9 | 3 | 0 | 1 | 3 | 19 | 0 | 1 | 1 | 0 |
| OM | Oman | 0 | 0 | 0 | 0 | 0 | 0 | 6 | 3 | 0 | 1 | 6 |
| PF | French Polynesia | 0 | 0 | 0 | 0 | 0 | 0 | 2 | 0 | 1 | 1 | 0 |
| PH | Philippines | 0 | 0 | 0 | 0 | 0 | 3 | 16 | 0 | 0 | 8 | 2 |
| PT | Portugal | 2 | 2 | 0 | 1 | 0 | 0 | 23 | 1 | 34 | 17 | 49 |
| PW | Palau | 3 | 1 | 1 | 0 | 0 | 1 | 538 | 19 | 58 | 45 | 59 |
| SE | Sweden | 0 | 1 | 0 | 0 | 0 | 0 | 11 | 3 | 5 | 5 | 7 |
| TH | Thailand | 71 | 36 | 18 | 96 | 3 | 107 | 2066 | 61 | 788 | 7683 | 1314 |
| TW | Taiwan | 1 | 0 | 1 | 0 | 0 | 0 | 50 | 3 | 30 | 10 | 2 |
| US | United States | 50 | 34 | 64 | 2 | 2 | 54 | 491 | 36 | 151 | 134 | 68 |
| ZA | South Africa | 121 | 14 | 5 | 0 | 3 | 4 | 26 | 2 | 14 | 0 | 17 |
|  | **Total** | **7482** | **1854** | **15011** | **601** | **1447** | **861** | **13251** | **720** | **4634** | **9849** | **3194** |

Table S4. Summary of total plastic counts based on colours by country.

| **Country ISO Alpha-2 code** | **Country** | **White** | **Clear or opaque** | **Black** | **Yellow** | **Green** | **Grey** | **Blue** | **Red** | **Pink** | **Orange** |
| --- | --- | --- | --- | --- | --- | --- | --- | --- | --- | --- | --- |
| AQ | Antarctica | 0 | 1 | 0 | 0 | 0 | 0 | 0 | 0 | 0 | 0 |
| AR | Argentina | 99 | 14 | 33 | 14 | 98 | 4 | 96 | 32 | 13 | 21 |
| AT | Austria | 4 | 4 | 1 | 0 | 0 | 0 | 3 | 2 | 1 | 0 |
| AU | Australia | 55 | 22 | 4 | 1 | 9 | 2 | 25 | 10 | 0 | 1 |
| BE | Belgium | 1 | 0 | 0 | 0 | 3 | 0 | 1 | 0 | 0 | 0 |
| BH | Bahrain | 51 | 12 | 56 | 43 | 12 | 2 | 25 | 3 | 2 | 6 |
| BR | Razil | 26 | 63 | 3 | 4 | 11 | 6 | 29 | 4 | 4 | 6 |
| CA | Canada | 3 | 1 | 3 | 0 | 0 | 0 | 2 | 1 | 0 | 0 |
| CV | Cabo Verde | 11 | 2 | 0 | 2 | 16 | 5 | 13 | 2 | 0 | 0 |
| EG | Egypt | 3 | 3 | 0 | 0 | 0 | 0 | 0 | 0 | 0 | 0 |
| ES | Spain | 25 | 34 | 16 | 8 | 27 | 4 | 20 | 7 | 5 | 9 |
| FK | Falkland Islands | 0 | 0 | 0 | 0 | 0 | 0 | 0 | 0 | 0 | 0 |
| FR | France | 8 | 2 | 0 | 1 | 0 | 0 | 1 | 0 | 0 | 0 |
| GB | Great Britain | 1179 | 3389 | 1489 | 258 | 526 | 585 | 1013 | 368 | 94 | 77 |
| GR | Greece | 101 | 145 | 12 | 18 | 21 | 5 | 93 | 11 | 10 | 1 |
| GS | South Georgia | 0 | 0 | 0 | 0 | 0 | 0 | 0 | 0 | 0 | 0 |
| HK | Hong Kong | 35 | 21 | 7 | 5 | 2 | 0 | 3 | 17 | 0 | 2 |
| HN | Honduras | 213 | 257 | 13 | 8 | 42 | 17 | 100 | 11 | 11 | 17 |
| ID | Indonesia | 42 | 48 | 25 | 6 | 25 | 1 | 22 | 5 | 2 | 5 |
| IE | Ireland | 0 | 0 | 0 | 0 | 0 | 0 | 0 | 0 | 0 | 0 |
| IN | India | 52 | 6 | 6 | 13 | 28 | 4 | 43 | 8 | 6 | 1 |
| IT | Italy | 41 | 62 | 11 | 14 | 20 | 1 | 48 | 2 | 2 | 3 |
| KE | Kenya | 201 | 1 | 3 | 3 | 54 | 10 | 72 | 6 | 0 | 0 |
| MT | Malta | 8 | 2 | 0 | 1 | 0 | 0 | 4 | 0 | 0 | 0 |
| MX | Mexico | 143 | 177 | 17 | 10 | 24 | 4 | 49 | 3 | 6 | 4 |
| MY | Malaysia | 262 | 259 | 17 | 43 | 143 | 13 | 213 | 38 | 33 | 19 |
| MZ | Mozambique | 2714 | 1680 | 339 | 206 | 558 | 164 | 1188 | 55 | 138 | 78 |
| NL | Netherlands | 18293 | 512 | 826 | 5 | 27 | 53 | 59 | 22 | 3 | 17 |
| NZ | New Zealand | 19 | 0 | 0 | 3 | 5 | 1 | 7 | 3 | 0 | 1 |
| OM | Oman | 2 | 0 | 0 | 1 | 0 | 0 | 4 | 1 | 1 | 0 |
| PF | French Polynesia | 2 | 1 | 0 | 0 | 0 | 0 | 0 | 0 | 0 | 0 |
| PH | Philippines | 2 | 1 | 1 | 1 | 2 | 0 | 9 | 1 | 0 | 2 |
| PT | Portugal | 20 | 3 | 1 | 3 | 15 | 2 | 10 | 7 | 1 | 1 |
| PW | Palau | 367 | 52 | 4 | 2 | 55 | 1 | 114 | 11 | 4 | 11 |
| ZA | Sweden | 107 | 30 | 8 | 2 | 5 | 8 | 13 | 2 | 0 | 14 |
| SE | Thailand | 15 | 0 | 1 | 0 | 0 | 1 | 1 | 1 | 1 | 0 |
| TH | Taiwan | 1025 | 1209 | 148 | 93 | 264 | 18 | 357 | 65 | 42 | 25 |
| TW | United States | 33 | 2 | 2 | 4 | 24 | 1 | 18 | 0 | 0 | 1 |
| US | South Africa | 262 | 227 | 60 | 39 | 86 | 21 | 132 | 19 | 14 | 24 |
|  | **Total** | **25424** | **8242** | **3106** | **811** | **2102** | **933** | **3787** | **717** | **393** | **346** |

Table S5. Average counts per 0.005 m^3^ of sediment for different types of MP and MEPs for each country.

| **ISO Alpha-2 country code** | **Country** | **Nurdle Spherical** | **Nurdle cylindrical** | **Nurdle disk** | **Nurdle cube** | **Bio-beads** | **Primary microplastics** | **Secondary microplastics** | **Primary mesoplastics** | **Secondary mesoplastics** | **Expanded Polystyrene (EPS) Balls** | **Expanded Polystyrene (EPS) Pieces** | **Totals** |
| --- | --- | --- | --- | --- | --- | --- | --- | --- | --- | --- | --- | --- | --- |
| AQ | Antarctica | 0.0 | 0.0 | 0.0 | 0.0 | 0.0 | 0.0 | 0.1 | 0.0 | 0.0 | 0.0 | 0.0 | **0.1** |
| AR | Argentina | 0.0 | 0.1 | 0.0 | 0.0 | 0.0 | 0.3 | 3.7 | 0.1 | 3.5 | 1.6 | 0.8 | **9.9** |
| AT | Austria | 0.0 | 0.0 | 0.0 | 0.0 | 0.0 | 0.0 | 5.0 | 3.0 | 7.0 | 0.0 | 1.0 | **16.0** |
| AU | Australia | 0.8 | 1.4 | 1.1 | 0.2 | 0.2 | 0.2 | 3.5 | 0.3 | 2.2 | 0.5 | 0.0 | **10.4** |
| BE | Belgium | 0.0 | 0.0 | 0.0 | 0.0 | 0.0 | 1.5 | 0.5 | 0.5 | 0.0 | 0.0 | 0.0 | **2.5** |
| BH | Bahrain | 0.1 | 0.3 | 0.0 | 0.0 | 0.3 | 0.9 | 1.5 | 0.7 | 3.1 | 0.2 | 0.2 | **7.2** |
| BR | Razil | 15.7 | 0.0 | 3.3 | 0.0 | 0.7 | 10.7 | 18.0 | 2.7 | 1.0 | 0.3 | 2.0 | **54.3** |
| CA | Canada | 0.0 | 0.0 | 0.0 | 0.0 | 0.2 | 0.7 | 0.8 | 0.0 | 0.0 | 0.0 | 0.2 | **1.8** |
| CV | Cabo Verde | 0.7 | 0.0 | 0.0 | 0.0 | 0.0 | 0.0 | 4.7 | 0.3 | 11.3 | 0.0 | 0.0 | **17.0** |
| EG | Egypt | 0.3 | 0.0 | 0.0 | 0.0 | 0.0 | 0.0 | 1.3 | 0.0 | 0.3 | 0.0 | 0.0 | **2.0** |
| ES | Spain | 1.2 | 2.0 | 1.3 | 0.7 | 0.0 | 2.7 | 12.0 | 0.5 | 5.5 | 0.0 | 0.5 | **26.3** |
| FK | Falkland Islands | 0.0 | 0.0 | 0.0 | 0.0 | 0.0 | 0.0 | 0.0 | 0.0 | 0.0 | 0.0 | 0.0 | **0.0** |
| FR | France | 0.3 | 0.7 | 0.3 | 0.0 | 0.0 | 0.3 | 2.3 | 0.0 | 0.0 | 4.0 | 0.0 | **8.0** |
| GB | Great Britain | 1.7 | 4.2 | 6.0 | 0.9 | 1.6 | 0.3 | 8.2 | 0.3 | 3.8 | 2.2 | 2.4 | **31.6** |
| GR | Greece | 0.5 | 2.0 | 7.0 | 0.0 | 0.0 | 0.0 | 194.5 | 0.0 | 4.5 | 3.5 | 9.0 | **221.0** |
| GS | South Georgia | 0.0 | 0.0 | 0.0 | 0.0 | 0.0 | 0.0 | 0.0 | 0.0 | 0.0 | 0.0 | 0.0 | **0.0** |
| HK | Hong Kong | 18.1 | 0.3 | 0.0 | 0.5 | 0.8 | 0.0 | 1.5 | 1.3 | 0.5 | 8.3 | 6.5 | **37.8** |
| HN | Honduras | 35.0 | 17.0 | 3.5 | 4.5 | 3.5 | 1.0 | 199.0 | 0.0 | 81.0 | 1.5 | 18.5 | **364.5** |
| ID | Indonesia | 0.4 | 0.1 | 0.0 | 0.0 | 0.0 | 0.0 | 10.9 | 0.3 | 1.3 | 24.5 | 11.0 | **48.4** |
| IE | Ireland | 0.0 | 0.0 | 0.0 | 0.0 | 0.0 | 0.0 | 0.0 | 0.0 | 0.0 | 0.0 | 0.0 | **0.0** |
| IN | India | 2.1 | 0.0 | 0.0 | 0.0 | 0.1 | 1.7 | 1.8 | 0.7 | 4.0 | 0.1 | 0.4 | **10.9** |
| IT | Italy | 0.0 | 1.8 | 0.5 | 0.0 | 0.0 | 0.1 | 6.1 | 0.4 | 3.1 | 1.1 | 3.1 | **16.2** |
| KE | Kenya | 1.0 | 9.0 | 0.0 | 0.0 | 0.0 | 3.0 | 337.0 | 0.0 | 0.0 | 0.0 | 0.0 | **350.0** |
| MT | Malta | 0.0 | 0.0 | 0.0 | 0.0 | 0.0 | 0.5 | 7.0 | 0.0 | 0.0 | 6.0 | 1.5 | **15.0** |
| MX | Mexico | 0.0 | 0.0 | 0.0 | 0.0 | 0.0 | 0.1 | 8.5 | 0.4 | 2.6 | 0.1 | 0.2 | **11.7** |
| MY | Malaysia | 0.9 | 0.4 | 0.2 | 0.0 | 0.0 | 2.2 | 7.2 | 1.3 | 3.1 | 9.6 | 5.9 | **30.8** |
| MZ | Mozambique | 2.8 | 1.5 | 1.0 | 1.4 | 0.4 | 2.5 | 39.6 | 1.6 | 11.2 | 0.2 | 1.0 | **63.1** |
| NL | Netherlands | 274.2 | 1.7 | 579.3 | 0.6 | 37.3 | 0.3 | 0.3 | 6.8 | 0.3 | 0.0 | 0.1 | **900.8** |
| NZ | New Zealand | 0.3 | 0.9 | 0.3 | 0.0 | 0.1 | 0.3 | 1.9 | 0.0 | 0.1 | 0.1 | 0.0 | **4.0** |
| OM | Oman | 0.0 | 0.0 | 0.0 | 0.0 | 0.0 | 0.0 | 6.0 | 3.0 | 0.0 | 1.0 | 6.0 | **16.0** |
| PF | French Polynesia | 0.0 | 0.0 | 0.0 | 0.0 | 0.0 | 0.0 | 1.0 | 0.0 | 0.5 | 0.5 | 0.0 | **2.0** |
| PH | Philippines | 0.0 | 0.0 | 0.0 | 0.0 | 0.0 | 1.5 | 8.0 | 0.0 | 0.0 | 4.0 | 1.0 | **14.5** |
| PT | Portugal | 0.5 | 0.5 | 0.0 | 0.3 | 0.0 | 0.0 | 5.8 | 0.3 | 8.5 | 4.3 | 12.3 | **32.3** |
| PW | Palau | 0.2 | 0.1 | 0.1 | 0.0 | 0.0 | 0.1 | 28.3 | 1.0 | 3.1 | 2.4 | 3.1 | **38.2** |
| SE | Sweden | 0.0 | 0.5 | 0.0 | 0.0 | 0.0 | 0.0 | 5.5 | 1.5 | 2.5 | 2.5 | 3.5 | **16.0** |
| TH | Thailand | 0.4 | 0.2 | 0.1 | 0.5 | 0.0 | 0.6 | 11.0 | 0.3 | 4.2 | 40.9 | 7.0 | **65.1** |
| TW | Taiwan | 0.2 | 0.0 | 0.2 | 0.0 | 0.0 | 0.0 | 10.0 | 0.6 | 6.0 | 2.0 | 0.4 | **19.4** |
| US | United States | 0.6 | 0.4 | 0.8 | 0.0 | 0.0 | 0.7 | 6.1 | 0.5 | 1.9 | 1.7 | 0.9 | **13.6** |
| ZA | South Africa | 24.2 | 2.8 | 1.0 | 0.0 | 0.6 | 0.8 | 5.2 | 0.4 | 2.8 | 0.0 | 3.4 | **41.2** |
|  | **Totals** | **382.2** | **47.7** | **605.9** | **9.6** | **45.8** | **32.8** | **963.9** | **28.6** | **178.7** | **122.8** | **101.5** |  |

Table S6. MP and MEP colour data showing average counts per 0.005 m^3^ of sediment collected in each country.

| **ISO Alpha-2 country code** | **Country** | **White** | **Clear or opaque** | **Black** | **Yellow** | **Green** | **Grey** | **Blue** | **Red** | **Pink** | **Orange** | **Totals** |
| --- | --- | --- | --- | --- | --- | --- | --- | --- | --- | --- | --- | --- |
| AQ | Antarctica | 0.0 | 0.1 | 0.0 | 0.0 | 0.0 | 0.0 | 0.0 | 0.0 | 0.0 | 0.0 | 0.1 |
| AR | Argentina | 4.1 | 0.3 | 0.6 | 0.3 | 1.8 | 0.1 | 1.7 | 0.6 | 0.2 | 0.4 | 10.0 |
| AT | Austria | 5.0 | 4.0 | 1.0 | 0.0 | 0.0 | 0.0 | 3.0 | 2.0 | 1.0 | 0.0 | 16.0 |
| AU | Australia | 4.7 | 1.7 | 0.3 | 0.1 | 0.7 | 0.2 | 1.9 | 0.8 | 0.0 | 0.1 | 10.4 |
| BE | Belgium | 0.5 | 0.0 | 0.0 | 0.0 | 1.5 | 0.0 | 0.5 | 0.0 | 0.0 | 0.0 | 2.5 |
| BH | Bahrain | 2.0 | 0.4 | 1.8 | 1.4 | 0.4 | 0.1 | 0.8 | 0.1 | 0.1 | 0.2 | 7.2 |
| BR | Razil | 11.0 | 21.0 | 1.0 | 1.3 | 3.7 | 2.0 | 9.7 | 1.3 | 1.3 | 2.0 | 54.3 |
| CA | Canada | 0.7 | 0.2 | 0.5 | 0.0 | 0.0 | 0.0 | 0.3 | 0.2 | 0.0 | 0.0 | 1.8 |
| CV | Cabo Verde | 3.7 | 0.7 | 0.0 | 0.7 | 5.3 | 1.7 | 4.3 | 0.7 | 0.0 | 0.0 | 17.0 |
| EG | Egypt | 1.0 | 1.0 | 0.0 | 0.0 | 0.0 | 0.0 | 0.0 | 0.0 | 0.0 | 0.0 | 2.0 |
| ES | Spain | 4.7 | 5.7 | 2.7 | 1.3 | 4.5 | 0.7 | 3.3 | 1.2 | 0.8 | 1.5 | 26.3 |
| FK | Falkland Islands | 0.0 | 0.0 | 0.0 | 0.0 | 0.0 | 0.0 | 0.0 | 0.0 | 0.0 | 0.0 | 0.0 |
| FR | France | 6.7 | 0.7 | 0.0 | 0.3 | 0.0 | 0.0 | 0.3 | 0.0 | 0.0 | 0.0 | 8.0 |
| GB | Great Britain | 8.1 | 10.2 | 4.5 | 0.8 | 1.6 | 1.8 | 3.0 | 1.1 | 0.3 | 0.2 | 31.6 |
| GR | Greece | 63.0 | 72.5 | 6.0 | 9.0 | 10.5 | 2.5 | 46.5 | 5.5 | 5.0 | 0.5 | 221.0 |
| GS | South Georgia | 0.0 | 0.0 | 0.0 | 0.0 | 0.0 | 0.0 | 0.0 | 0.0 | 0.0 | 0.0 | 0.0 |
| HK | Hong Kong | 23.5 | 5.3 | 1.8 | 1.3 | 0.5 | 0.0 | 0.8 | 4.3 | 0.0 | 0.5 | 37.8 |
| HN | Honduras | 126.5 | 128.5 | 6.5 | 4.0 | 21.0 | 8.5 | 50.0 | 5.5 | 5.5 | 8.5 | 364.5 |
| ID | Indonesia | 38.5 | 3.4 | 1.8 | 0.4 | 1.8 | 0.1 | 1.6 | 0.4 | 0.1 | 0.4 | 48.4 |
| IE | Ireland | 0.0 | 0.0 | 0.0 | 0.0 | 0.0 | 0.0 | 0.0 | 0.0 | 0.0 | 0.0 | 0.0 |
| IN | India | 3.7 | 0.4 | 0.4 | 0.8 | 1.8 | 0.3 | 2.7 | 0.5 | 0.4 | 0.1 | 10.8 |
| IT | Italy | 6.6 | 3.7 | 0.7 | 0.8 | 1.2 | 0.1 | 2.8 | 0.1 | 0.1 | 0.2 | 16.2 |
| KE | Kenya | 201.0 | 1.0 | 3.0 | 3.0 | 54.0 | 10.0 | 72.0 | 6.0 | 0.0 | 0.0 | 350.0 |
| MT | Malta | 11.5 | 1.0 | 0.0 | 0.5 | 0.0 | 0.0 | 2.0 | 0.0 | 0.0 | 0.0 | 15.0 |
| MX | Mexico | 4.0 | 4.7 | 0.5 | 0.3 | 0.6 | 0.1 | 1.1 | 0.1 | 0.2 | 0.1 | 11.7 |
| MY | Malaysia | 19.3 | 3.8 | 0.3 | 0.6 | 2.1 | 0.2 | 3.1 | 0.6 | 0.5 | 0.3 | 30.8 |
| MZ | Mozambique | 24.8 | 14.6 | 3.0 | 1.8 | 4.9 | 1.4 | 10.3 | 0.5 | 1.2 | 0.7 | 63.1 |
| NL | Netherlands | 831.6 | 23.3 | 37.6 | 0.2 | 1.2 | 2.4 | 2.7 | 1.0 | 0.1 | 0.8 | 900.8 |
| NZ | New Zealand | 2.0 | 0.0 | 0.0 | 0.3 | 0.5 | 0.1 | 0.7 | 0.3 | 0.0 | 0.1 | 4.0 |
| OM | Oman | 9.0 | 0.0 | 0.0 | 1.0 | 0.0 | 0.0 | 4.0 | 1.0 | 1.0 | 0.0 | 16.0 |
| PF | French Polynesia | 1.5 | 0.5 | 0.0 | 0.0 | 0.0 | 0.0 | 0.0 | 0.0 | 0.0 | 0.0 | 2.0 |
| PH | Philippines | 6.0 | 0.5 | 0.5 | 0.5 | 1.0 | 0.0 | 4.5 | 0.5 | 0.0 | 1.0 | 14.5 |
| PT | Portugal | 21.5 | 0.8 | 0.3 | 0.8 | 3.8 | 0.5 | 2.5 | 1.8 | 0.3 | 0.3 | 32.3 |
| PW | Palau | 24.8 | 2.7 | 0.2 | 0.1 | 2.9 | 0.1 | 6.0 | 0.6 | 0.2 | 0.6 | 38.2 |
| SE | Sweden | 13.5 | 0.0 | 0.5 | 0.0 | 0.0 | 0.5 | 0.5 | 0.5 | 0.5 | 0.0 | 16.0 |
| TH | Thailand | 53.3 | 6.4 | 0.8 | 0.5 | 1.4 | 0.1 | 1.9 | 0.4 | 0.2 | 0.1 | 65.1 |
| TW | Taiwan | 9.0 | 0.4 | 0.4 | 0.8 | 4.8 | 0.2 | 3.6 | 0.0 | 0.0 | 0.2 | 19.4 |
| US | United States | 5.8 | 2.8 | 0.8 | 0.5 | 1.1 | 0.3 | 1.7 | 0.2 | 0.2 | 0.3 | 13.6 |
| ZA | South Africa | 24.8 | 6.0 | 1.6 | 0.4 | 1.0 | 1.6 | 2.6 | 0.4 | 0.0 | 2.8 | 41.2 |
|  | **Totals** | 1577.2 | 328.0 | 78.6 | 33.7 | 135.4 | 35.2 | 252.7 | 37.8 | 19.2 | 21.7 |  |

Table S7. Regional data based on submissions

| **ISO Alpha-2 country code** | **Country** | **Regions** | **Total number of samples** |
| --- | --- | --- | --- |
| AQ | Antarctica | Deception Island  South Shetland Islands  Antarctic Peninsula | 3  2  2 |
| AR | Argentina | Chubut  Tierra del Fuego  Rio Negro | 53  2  1 |
| AT | Austria | Styria | 1 |
| AU | Australia | Tasmania  Victoria  New South Wales  Western Australia | 4  4  3  2 |
| BE | Belgium | West Flanders | 2 |
| BH | Bahrain | Northern  Muharraq  Capital | 16  8  7 |
| BR | Brazil | Alagoas  Santa Caterina  Sao Paulo | 1  1  1 |
| CA | Canada | British Columbia  Manitoba | 4  2 |
| CV | Cabo Verde | Sal | 3 |
| EG | Egypt | El Qoosier | 3 |
| ES | Spain | Murcia  Biscay  Lanzarote | 3  2  1 |
| FK | Falkland Islands | West Falkland | 1 |
| FR | France | Brittany  Cote D’Azur | 2  1 |
| GB | Great Britain | West Sussex  Cornwall  Hampshire  North Yorkshire  Norfolk  Devon  Dorset  Durham  East Riding  Anglesey  Highlands  Glamorgan  Cardiganshire  Isle of Wight  Pembrokeshire  Fife  Orkney  Northumberland  Kent  Merseyside  Wiltshire  Lothian  Argyle and Bute  South Ayrshire  County Down | 148  30  27  25  23  14  8  8  8  8  5  4  4  3  3  3  3  2  1  1  1  1  1  1  1 |
| GR | Greece | Leipsoi  Trikeri | 1  1 |
| GS | South Georgia | South Georgia Island | 1 |
| HK | Hong Kong | Hong Kong | 4 |
| HN | Honduras | Roatan Island  Utila Island | 1  1 |
| ID | Indonesia | Gilli Trawangan  North Sulawesi | 13  1 |
| IE | Ireland | Galway | 1 |
| IN | India | Kerala  Karnataka | 11  5 |
| IT | Italy | Sicily  Sardinia | 13  4 |
| KE | Kenya | Kilifi | 1 |
| MT | Malta | Malta | 2 |
| MX | Mexico | Baja California  Nayarit  Quintana Roo | 36  1  1 |
| MY | Malaysia | Pulau Tengah  Pulau Besar  Pulau Menserip  Pulau Harimau  Pulau Hujong  Pulau Gual  Rawa Island  Pulau Seri  Pulau Rawa | 20  15  8  7  6  5  4  2  1 |
| MZ | Mozambique | Inhambane  Maputo | 60  55 |
| NL | Netherlands | Groningen  Zeeland  The Hague | 19  2  1 |
| NZ | New Zealand | Otago  Wellington  Gisborne | 7  2  1 |
| OM | Oman | Muscat | 1 |
| PF | French Polynesia | Tahiti | 2 |
| PH | Philippines | Mindoro | 2 |
| PT | Portugal | Centro  Algarve  Lisbon | 2  1  1 |
| PW | Palau | Ngaraard  Ngeaur  Malakal  Melekeok  Omekang | 13  2  1  2  1 |
| SE | Sweden | Stockholm | 2 |
| TH | Thailand | Koh Tao island  Phangnga | 155  33 |
| TW | Taiwan | Taitung  Anping  Hsinchu  Qingshui | 2  1  1  1 |
| US | United States | Maine  California  Massachusetts  Michigan  Texas  Washington  Minnesota  Ohio  Hawaii  New York  Oregon  Connecticut  New Hampshire  North Carolina  Wisconsin | 14  11  10  8  8  7  5  5  3  2  2  2  1  1  1 |
| ZA | South Africa | Western Cape  KwaZulu Natal | 4  1 |
|  |  | **Total regions 123** | **Total samples 1089** |
